# Stiff and tough PDMS-MMT layered nanocomposites visualized by AIE luminogens

**DOI:** 10.1038/s41467-021-24835-w

**Published:** 2021-07-27

**Authors:** Jingsong Peng, Antoni P. Tomsia, Lei Jiang, Ben Zhong Tang, Qunfeng Cheng

**Affiliations:** 1 School of Chemistry, Key Laboratory of Bio-inspired Smart Interfacial Science and Technology of Ministry of Education, Beijing Advanced Innovation Center for Biomedical Engineering, Beihang University, Beijing, China; 2 Department of Chemistry, The Hong Kong Branch of Chinese National Engineering Research Center for Tissue Restoration and Reconstruction, Institute of Molecular Functional Materials, Division of Life Science and State Key Laboratory of Molecular Neuroscience, The Hong Kong University of Science and Technology, Clear Water Bay, Kowloon, Hong Kong China; 3 School of Materials Science and Engineering, Zhengzhou University, Zhengzhou, China

**Keywords:** Polymers, Bioinspired materials, Structural properties

## Abstract

Polydimethylsiloxane (PDMS) is a widely used soft material that exhibits excellent stability and transparency. But the difficulty of fine-tuning its Young’s modulus and its low toughness significantly hinder its application in fields such as tissue engineering and flexible devices. Inspired by nacre, here we report on the development of PDMS-montmorillonite layered (PDMS-MMT-L) nanocomposites via the ice-templating technique, resulting in 23 and 12 times improvement in Young’s modulus and toughness as compared with pure PDMS. Confocal fluorescence microscopy assisted by aggregation-induced emission (AIE) luminogens reveals three-dimensional reconstruction and in situ crack tracing of the nacre-inspired PDMS-MMT-L nanocomposite. The PDMS-MMT-L nanocomposite is toughened with mechanisms such as crack deflection and bridging. The AIE-assisted visualization of the crack propagation for nacre-inspired layered nanocomposites provides an advanced and universal characterization technique for organic-inorganic nanocomposites.

## Abstract (web summary)

Fine tuning of mechanical properties in elastomers is important for application of elastomers in flexible devices and biomedical field. Here, the authors prepare a nacre inspired PDMS-montmorrillonite composite with good mechanical properties and demonstrate crack tracing using aggregation-induced emission luminogens.

## Introduction

Polydimethylsiloxane (PDMS), one of the most widely used soft materials due to its excellent biocompatibility and stability^1^, high transparency^2^, and easy moldability^3^, demonstrates promising applications in fields such as microfluidics, tissue engineering, flexible devices, wearable equipment, and many others^4–6^. However, PDMS exhibits a low Young’s modulus, and for many of these applications, improved approaches are needed to improve its stiffness and load-bearing capabilities^5,7–9^. Varying the density of crosslinking will effectively increase the Young’s modulus from 0.05 MPa to about 2 MPa^7,10^. But strong crosslinking may also lead to the impairment of PDMS’s stretchability. For example, a PDMS polymer network containing boroxine as the crosslinking agent boosts the Young’s modulus to a high value of 182 MPa, but the elongation at break is only ~10%^11^. In addition, the glass transition temperature (T_g_) also increases to a value of 65 °C, indicating that the PDMS-boroxine will not behave as an elastomer at room temperature^11^. In nature, however, stretchable materials with a high Young’s modulus are fairly common and include skin and leather, providing animals with the capability of unlimited movement as well as protection^12,13^.

Conventional PDMS possesses excellent stretchability while its toughness is 1–2 orders of magnitude lower than that of natural rubbers^14,15^. To the best of our knowledge, the current methods to toughen soft materials such as hydrogels and elastomers mainly involve introducing nanofillers^14,16,17^, constructing a double-network structure^18,19^, and designing a macroscale heterogeneous structure^10,20–23^. For various nanofillers, the interface between them and the soft polymer matrix plays a key role in the toughening effectiveness^14,16^. For example, silica nanoparticles, which can form physical crosslinking in the polymer matrix, are usually applied to toughen the poly(dimethylacrylamide) (PDMA) hydrogel^16^, resulting in eight times improvement of toughness. Recently, liquid metal particles were also used to toughen PDMS via crack deflection^14^, resulting in 50 times improvement of toughness. In addition, the toughness of elastomers or hydrogels can also be enhanced significantly through a double-network structure. For instance, the toughness of polyacrylic elastomer is boosted by two orders of magnitude due to the energy dissipation of sacrificial chains in double-network or triple-network structures^19^. Furthermore, constructing macroscale fiber-reinforced architecture can also toughen PDMS; the resulted toughness is comparable to that of natural rubbers^10^. The debonding between macroscale fibers and PDMS matrix leads to distinctive crack deflection. However, a considerable challenge is to enhance the Young’s modulus and toughness together of the aforementioned hydrogels or elastomers to the level of natural soft materials such as skin or leather^13^.

In nature, living things have developed materials that combine an extraordinary toughness and Young’s modulus, such as nacre with its “brick-and-mortar” structure^24–28^. The major component of nacre, aragonite, provides superior Young’s modulus and strength^29^. Abundant interface interactions effectively impede crack propagation, giving nacre excellent toughness^28,30^. For decades, numerous scientists described efforts to fabricate nanocomposites integrating high strength and toughness, mimicking the delicate structure of nacre^31–41^. However, it is difficult to reveal the toughening mechanism of nacre-inspired layered polymer nanocomposites by conventional characterization methods, such as in situ scanning electron microscopy (SEM). The disadvantages of conventional SEM are the lack of three-dimensional information, interference from the sample surface, and insufficient electrical conductivity of the samples.

With the discovery of aggregation-induced emission (AIE) luminogens^42,43^, the AIE-based imaging technique has made remarkable progress in the fields of biology and medicine^44^. Unlike traditional luminophores, AIE luminogens show strong fluorescence in the aggregative state, especially in the solid state. In addition, AIE molecules also have obvious advantages, such as high fluorescence intensity and excellent stability against photobleaching^45^. Thus, AIE-assisted confocal fluorescence microscopy (CFM) is an ideal method for characterizing the microstructure of polymer nanocomposites. For example, the three-dimensional (3D) dispersion of AIE-modified nanoclay in the polymer matrix was described using CFM^46^. A multilayered polymer composite with cracks was also imaged with CFM^47^. In addition, some previous works have been devoted to the characterization of in situ crack propagation on a sample surface coated with AIE luminogen^48–50^. However, imaging in situ crack propagation, especially in three dimensions with AIE-assisted CFM, which would directly demonstrate the toughening mechanism, was not achieved.

We constructed PDMS-montmorillonite layered (PDMS-MMT-L) nanocomposites inspired by nacre, resulting in 23 and 12 times improvement in Young’s modulus and toughness as compared with pure PDMS, respectively. The 3D structure of nacre-inspired PDMS-MMT-L nanocomposites was reconstructed using fluorescence images, acquired by the confocal imaging technique assisted by AIE molecules. The in situ tracing of crack propagation, as visualized by AIE, revealed that the substantial enhancement in Young’s modulus is owing to the continuous stiff MMT-based scaffold, which generates high stress at the beginning of the stretching process. The crack deflection and crack bridging induced by the nacre-inspired layered structure both lead to the increase of toughness. The AIE-assisted characterization technique can serve as a universal method to better evaluate stiffening and toughening mechanisms of organic-inorganic nanocomposites. In addition, the fabrication of the nacre-inspired layered PDMS-MMT nanocomposite provides an advanced research strategy for a stiffening and toughening mechanism of organic-inorganic nanocomposites.

## Results

### Fabrication of AIE-labeled PDMS-MMT layered nanocomposites

**Fig. 1:**
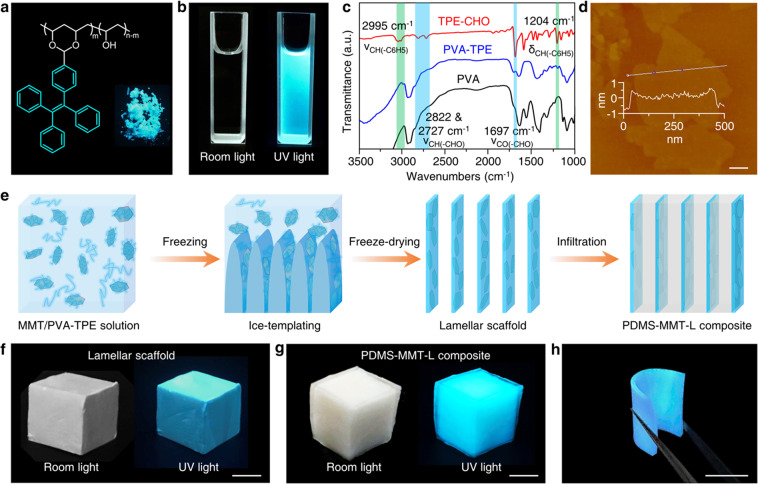
Fabrication of PDMS-MMT-L nanocomposites functionalized with AIE luminogen. **a** Molecule structure of PVA-TPE. **b** Fluorescence of PVA-TPE solution. **c** FTIR spectra of TPE-CHO (red), PVA-TPE (blue), and PVA (black). The disappearance of characteristic peaks of aldehyde group and the arising of characteristic peaks of phenyl groups on the PVA-TPE spectrum demonstrate the successful grafting process. **d** AFM image of MMT nanosheets shows a thickness of 0.75 nm. Scale bar: 100 nm. **e** Fabrication process of the nacre-inspired PDMS-MMT-L nanocomposite. The digital photos under room light and ultraviolet (UV) light of MMT-PVA scaffold (**f**) and PDMS-MMT-L nanocomposite (**g**). Scale bar: 10 mm. **h** The PDMS-MMT-L nanocomposite shows excellent flexibility as well as fluorescence. Scale bar: 5 mm.

Polyvinyl alcohol (PVA) decorated with AIE luminogens was first fabricated. A typical AIE molecular 4-(1,2,2-triphenylvinyl)-benzaldehyde (TPE-CHO) was used^51,52^ whose aldehyde group reacted with hydroxyl groups by aldolization. The TPE groups were grafted onto polyvinyl alcohol (PVA) to obtain PVA-TPE polymer with blue fluorescence (Fig. 1a). The PVA and TPE-CHO were dissolved in dimethyl sulfoxide and mixed to react with each other. Then the reaction mixture was poured into the acetone to precipitate PVA-TPE, followed by a washing process with acetone. The precipitated PVA-TPE shows good water solubility and emits strong fluorescence in water solution (Fig. 1b). Fourier transform infrared spectroscopy (FTIR) was used to determine the success of the reaction between PVA and TPE-CHO. As shown in Fig. 1c, the double peaks appearing at wavenumbers of 2882 and 2727 cm^−1^ and the peak at 1697 cm^−1^ are characteristic peaks of the aldehyde group in TPE-CHO. These characteristic peaks of the aldehyde group disappeared in the obtained PVA-TPE, and new phenyl peaks appeared at 2995 cm^−1^ and 1204 cm^−1^, indicating that the TPE-CHO was successfully grafted onto the PVA polymer chains. Using hydrogen nuclear magnetic resonance (^1^H NMR), we determined that the degree of labeling of TPE molecules is ~0.078 mol% (Supplementary Fig. 1a). We tried to increase the degree of labeling by increasing the amount of TPE-CHO to about 0.16 mol%. However, the degree of labeling was still about 0.082 mol% (Supplementary Fig. 1b) due to severe steric hindrance of the TPE group^53^. The TPE-decorated PVA shows stronger fluorescence emission than other non-AIE luminogens. We grafted a derivative of pyrene (Py), 1-pyrenecarboxaldehyde (Py-CHO), to PVA polymer chains (PVA-Py) via a similar process as PVA-TPE for comparison. The fluorescence of as-prepared PVA-Py was much weaker than PVA-TPE with the same degree of labeling (Supplementary Fig. 2). Our results show that the AIE luminogen can enhance its fluorescence emitting in aggregative state, while the non-AIE luminogen leads to an aggregation-caused quenching (ACQ) effect^45^. There is no fluorescence resonance energy transfer between Py and PVA-TPE (Supplementary Fig. 3 and 4)^54^.

Next, MMT, a natural clay, was exfoliated into nanosheets^55,56^. The thickness of MMT nanosheets was about 0.75 nm as characterized by atomic force microscope (AFM) (Fig. 1d). The PVA-TPE solution and the MMT solution were then blended (1:1 weight ratio) into a homogeneous solution, followed by bidirectional freezing. In this process, ice crystals formed parallel platelets by bidirectional temperature gradients and entrapped the PVA-TPE polymers and MMT nanosheets into a lamellar structure^37^. After freeze-drying, the ice was sublimated, leaving a lamellar MMT-PVA scaffold. Finally, PDMS was introduced into the lamellar scaffold to fill the voids through vacuum-assisted infiltration. Using this procedure, the nacre-inspired PDMS-MMT-L was obtained (Fig. 1e). Thermogravimetric analysis (TGA) revealed that the content of PDMS is about 91.2 wt% (Supplementary Fig. 5). The introduction of MMT improved the stability of the scaffold, minimizing the shrinkage and deformation during freeze-drying and subsequent infiltration processes. Due to the decoration with the AIE molecules, the resulting MMT-PVA scaffold and PDMS-MMT-L nanocomposite exhibited fluorescence (Fig. 1f, g) while retaining the flexibility of the PDMS-based materials (Fig. 1h). For comparison, we have also prepared a randomly blended sample of MMT-PVA particles in the PDMS matrix (PDMS-MMT-R) with similar PDMS content (Supplementary Fig. 5).

### Characterization of PDMS-MMT layered nanocomposites by CFM

**Fig. 2:**
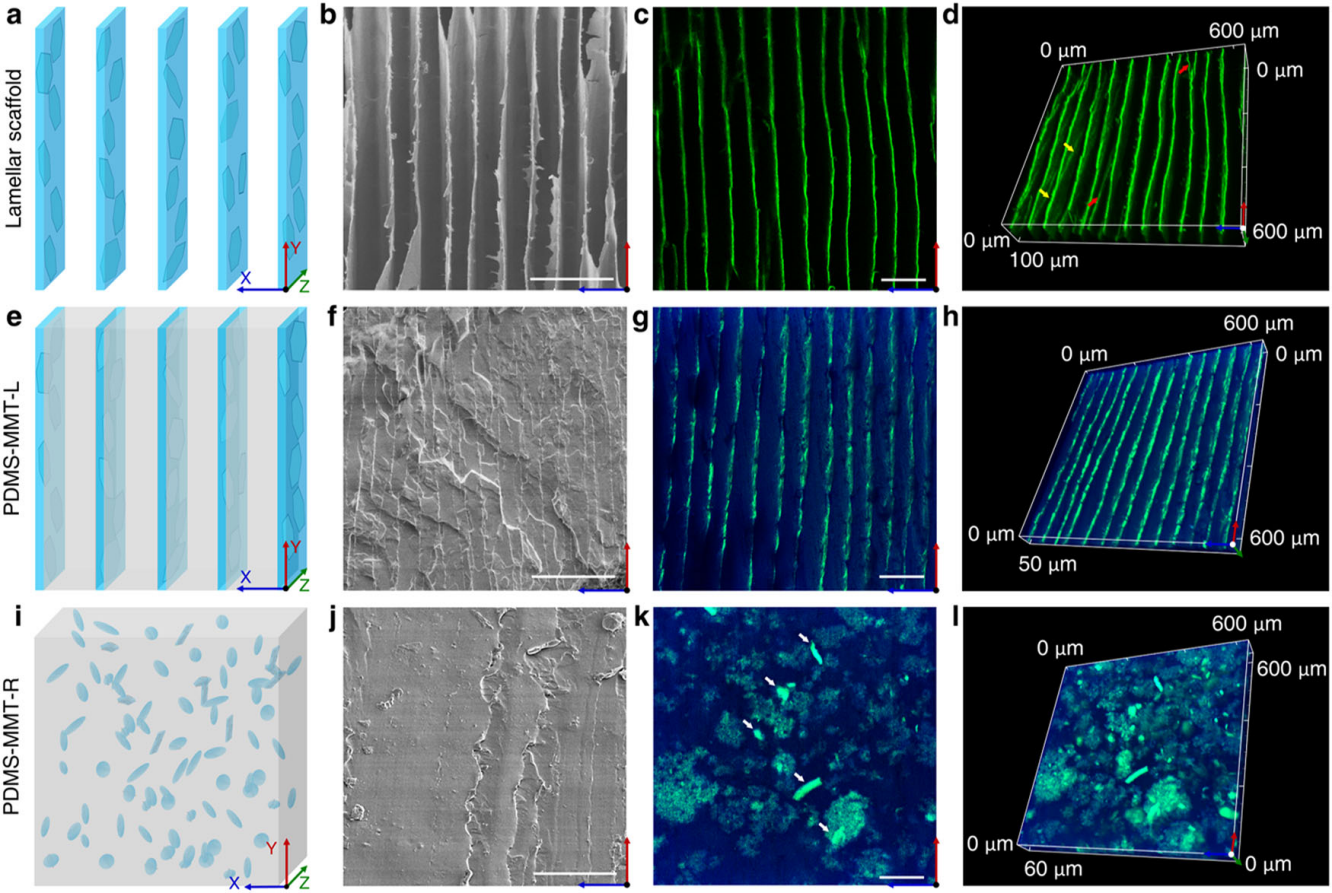
Imaging via SEM and AIE-assisted CFM. Schematic illustration (**a**), SEM image (**b**), and CFM image at the XY plane (**c**) as well as the 3D reconstruction (**d**) of the MMT-PVA scaffold. Schematic illustration (**e**), SEM image (**f**), and CFM image at the XY plane (**g**) as well as the 3D reconstruction (**h**) of PDMS-MMT-L nanocomposite. Schematic illustration (**i**), SEM image (**j**), and CFM image at the XY plane (**k**) as well as the 3D reconstruction (**l**) of PDMS-MMT-R nanocomposite. Scale bar: 100 μm.

Through the ice-templating process, the ordered MMT-PVA lamellar scaffold was constructed as shown in Fig. 2a. MMT nanosheets were embedded into PVA matrix forming a porous lamellar structure. The voids between adjacent layers were formed as the result of the sublimation of lamellar ice crystals. As illustrated in the side view (XY plane) of the layered scaffold (Fig. 2b), the interlayer spacing is about 30~50 μm, consistent with previously reported layered scaffolds made by ice-templating^35–38,57–59^. The CFM images were collected at different focal planes using the Z-scan technique. The influence of surface morphology on characterization and the fracture or deformation of MMT-PVA layers during sample preparation were ignored, leading to more exact structure information. As shown in Fig. 2c, ordered parallel stripes were distinguished in a 600 × 600 μm^2^ dark background, indicating a cross section of the lamellar scaffold on the XY plane. The layered structure acquired by CFM is much clearer than on the SEM image (Fig. 2b). Furthermore, the Z-axis scanning technology (step size: 1 μm) was conducted to obtain images of the XY plane at different depths (Supplementary Fig. 6), and accordingly, a 3D image of the layered scaffold was reconstructed (Fig. 2d). In addition to the layered structure of the scaffold, the bridges between the layers (red shears) and the ridges on the layer (yellow arrows) were also clearly observed from the 3D reconstruction. These bridges and ridges are special defects generated in the ice-templating process. The conventional SEM characterization for observing these bridges and edges requires samples with different viewing angles (Supplementary Fig. 7)^57^. Unfortunately, the bridges and edges were damaged due to the peeling or cutting of MMT-PVA layers during the sample preparation process.

For the nacre-inspired PDMS-MMT-L nanocomposite, PDMS was infiltrated into the voids of the lamellar scaffold (Fig. 2e). Parallel white strips, the pattern of the MMT-PVA lamellar scaffold, were observed in the SEM image in the XY plane (Fig. 2f). For the microscope analysis, conventional sample preparation processes, such as cutting or brittle fracture at low temperature, cannot make the surfaces smooth enough. When using fractured specimens, there exists extensive river-like morphology caused by a fracture during the sample preparation process, which interferes with the observation of an original layered structure. Furthermore, the scaffold and the matrix can be distinguished only based on different grayscale of the SEM image, showing only a darker phase (polymer) and a lighter phase (ceramics)^35^. As the scaffold layers are very thin compared with the matrix, it is difficult to determine whether the pattern is caused by fracture of the PDMS layers or during the cross-section preparation of the MMT-PVA scaffold.

Using CFM can effectively solve this problem. To distinguish the matrix and the scaffold, we added another fluorescent dye, 1-aminopyrene^60^, into the PDMS matrix. Using two-channel fluorescence imaging, we obtained a profile of the cross section in the XY plane of the PDMS-MMT-L nanocomposite. As shown in Fig. 2g, the matrix is labeled with blue while the scaffold is green, which effectively distinguishes each of them and avoids interference from surface morphology. It can be observed that the scaffold was tightly embedded into the matrix, separating the matrix into parallel layers. The interlayer spacing of the scaffold shows no substantial shrinkage. The 3D reconstruction (Fig. 2h) from CFM images in the XY plane at different depths (Supplementary Fig. 8) reveals no obvious gap between the scaffold and substrate, indicating sufficient infiltration of PDMS.

In the synthesis of PDMS-MMT-R nanocomposite (Fig. 2i), the MMT and PVA formed micrometer-scale particles due to extensive sonification that were homogeneously dispersed in PDMS matrix. The distribution of MMT-PVA particles cannot be revealed by SEM due to SEM’s difficulty in distinguishing between the matrix and fillers (Fig. 2j). The CFM image effectively distinguishes them by using different fluorescence labels. It can be observed that MMT-PVA particles were uniformly dispersed in the matrix (Fig. 2k), but there are still some distinct agglomerated particles visible (white arrows). Because of the hydrophilicity of MMT and PVA, they tend to aggregate in the hydrophobic PDMS matrix. A 3D reconstruction (Fig. 2l) from CFM images in the XY plane at different depths (Supplementary Fig. 9) also shows the spatial distribution of the fillers.

### Mechanical properties of PDMS-MMT layered nanocomposites

**Fig. 3:**
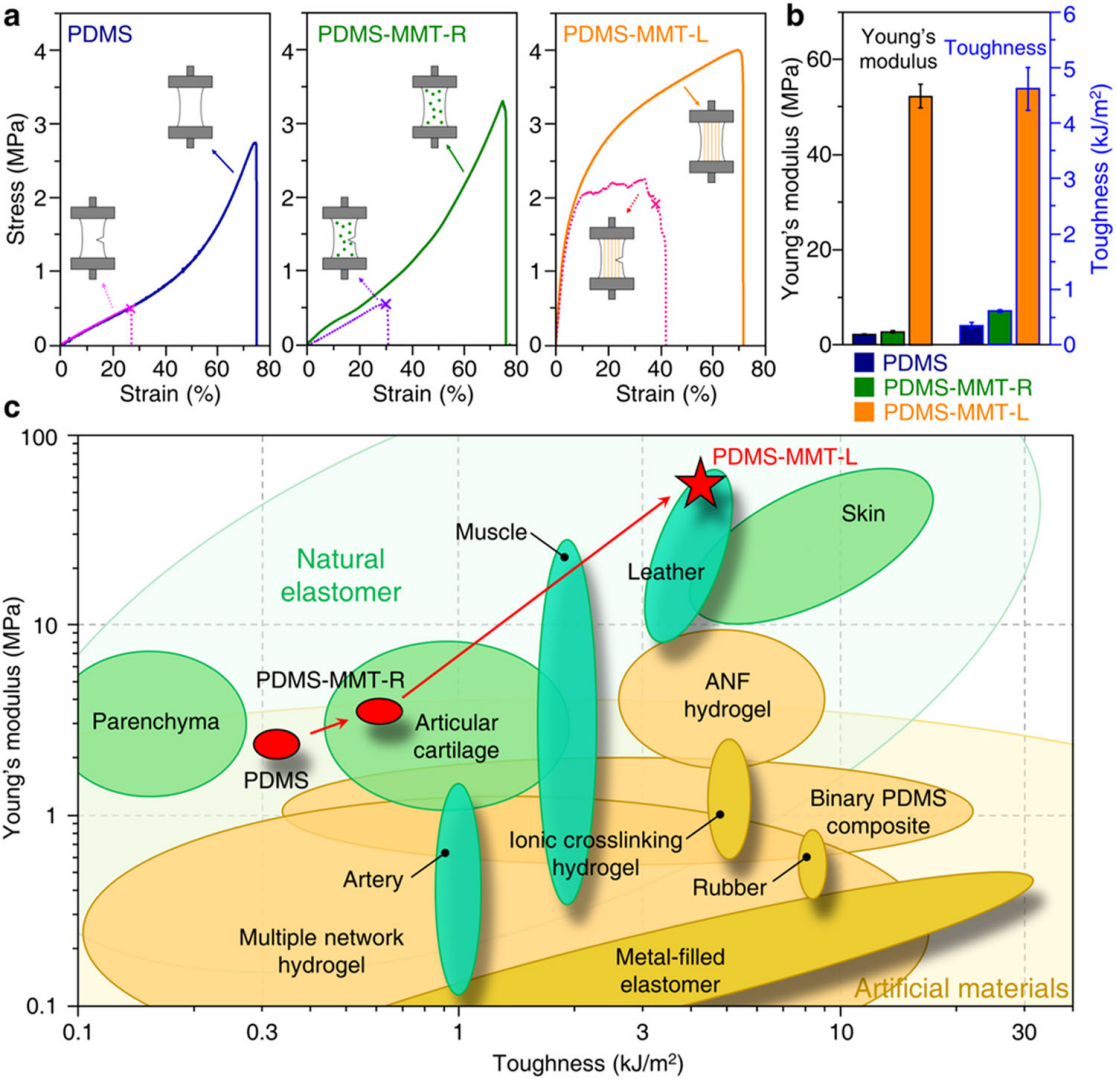
Mechanical properties of the PDMS-MMT-L nanocomposite. **a** Stress-strain curves of pure PDMS, PDMS-MMT-R, and PDMS-MMT-L nanocomposites. Dashed lines represent for the notched samples. **b** Comparison of the Young’s modulus and toughness among pure PDMS, PDMS-MMT-R, and PDMS-MMT-L nanocomposites. Error bars are mean ± SD. **c** Comparison of toughness and Young’s modulus among pure PDMS, PDMS-MMT-R, PDMS-MMT-L nanocomposite, and some artificial and natural soft materials. PDMS-MMT-L boosts the mechanical properties to a level comparable to natural materials.

Due to the unique architecture of the PDMS-MMT-L nanocomposite, its mechanical properties have been effectively improved, especially the Young’s modulus and toughness. As shown in Fig. 3a, the stress-strain curve of PDMS shows a typical pattern of elastomer^10,14^, with a Young’s modulus of 2.2 ± 0.2 MPa (Fig. 3b). Although PDMS possesses excellent elasticity and flexibility^4,5^, the crack resistance, or toughness, of PDMS is relatively poor^14,15^. Once a defect exists in PDMS, the strain at fracture will decrease sharply, as shown by the dotted line in Fig. 3a. The strain of the intact PDMS sample is 74%, while the strain of the notched sample drops to 27%, and the corresponding toughness is only 0.36 ± 0.05 kJ/m^2^. After introducing MMT-PVA particles, the Young’s modulus of PDMS-MMT-R is only slightly increased to 3.0 ± 0.3 MPa. This slight increase of Young’s modulus by randomly mixed particles fits well with the Guth-Gold model, which indicates that the fillers weakly interact with the rubbery matrix^16,61,62^. In addition, the toughness is also increased to 0.62 ± 0.05 kJ/m^2^ due to the energy dissipation by the weaker and breakable interactions between the fillers and the PDMS matrix^16^. The Young’s modulus of PDMS-MMT-L nanocomposite with MMT-PVA lamellar scaffold, however, reaches 52.3 ± 2.5 MPa, which is 23 times higher than that of pure PDMS. The toughness is enhanced to 4.6 ± 0.4 kJ/m^2^, 12 times higher than that of pure PDMS (Fig. 3b). Due to the anisotropic structure of PDMS-MMT-L, the mechanical properties differ when the direction of tension is changed. As shown in Supplementary Fig. 10, the Young’s modulus and toughness dramatically decrease to 6.1 ± 0.6 MPa and 0.11 ± 0.04 kJ/m^2^, respectively, when the tension is perpendicular to the layered scaffold. This behavior is typical of most biological materials.

It should be noted that the addition of AIE luminogen does not significantly influence the mechanical properties. As shown in Supplementary Fig. 11, we compared the unlabeled PDMS-MMT layered nanocomposite (PDMS-MMT-U) with the PDMS-MMT-L. They both show similar Young’s modulus and toughness. However, when we used other traditional hydrophilic luminophores, such as rhodamine B (RhB), the mechanical behavior was seriously affected. We fabricated RhB-labeled PDMS-MMT layered nanocomposite (PDMS-MMT-RhB), and the results indicate that the PDMS-MMT-RhB shows a more brittle behavior with an elongation at break of about only 30%, compared with PDMS-MMT-U (Supplementary Fig. 12a). This can be explained by the stiffening of MMT-PVA film with the addition of RhB as shown in Supplementary Fig. 12b. Thus, the AIE luminogen meets the most important requirement of fluorescence detection, that the luminogen should not affect the mechanical properties of samples.

We have also investigated the influence of the ratio of MMT to PVA on the mechanical properties. As shown in Supplementary Fig. 13a, the Young’s modulus of layered nanocomposites was improved from 35.1 ± 8.3 MPa to 117.2 ± 31.9 MPa when the weight ratio of MMT to PVA increased from 1:3 (PDMS-MMT-I) to 3:1 (PDMS-MMT-II). The toughness, however, was impaired at both higher and lower MMT content. Higher MMT content embrittled the nanocomposite, leading to a toughness of 2.9 ± 0.5 kJ/m^2^, while lower MMT content also reduced the toughness to only 0.27 ± 0.07 kJ/m^2^. Furthermore, we investigated the impact of interlayer spacing, as shown in Supplementary Fig. 13b. We controlled the freezing rate to achieve different interlayer spacings of 15–40 μm (PDMS-MMT-III), 30–50 μm (PDMS-MMT-L), and 70–160 μm (PDMS-MMT-IV) as shown in Supplementary Fig. 14. The results demonstrate that the Young’s modulus was very stable with different interlayer spacings. But the toughness declined to 0.71 ± 0.15 kJ/m^2^ with the increased interlayer spacing (70~160 μm). All the curves of pure PDMS, PDMS-MMT-L, PDMS-MMT-R, PDMS-MMT-I~IV, PDMS-MMT, PDMS-MMT-RhB, and various MMT-PVA films are listed in Supplementary Figs. 15–17.

Due to the simultaneous promotion of Young’s modulus and toughness, the mechanical properties of PDMS-MMT-L are comparable to some natural soft materials such as skin^12,13^, cartilage^63^, and muscle^64^. Figure 3c shows the Young’s modulus and toughness of some artificial soft materials, including hydrogels and elastomers^10,14,18,19,63^, as well as some natural soft materials, such as skin and cartilage^13,63,64^. Although the toughness of artificial materials is comparable to or even better than that of natural materials^14^, their Young’s moduli are inferior to those of natural materials. This is mainly because the current toughening strategies utilize conventional nanofillers: materials with a relatively low Young’s moduli^10,14^. For example, with PDMS toughened by liquid metal particles^14^, these particles will easily be elongated along the stretching direction. The crack is therefore prevented from propagating perpendicularly to the stretching direction, which induces the deflection of the crack and leads to the improvement of toughness. However, the liquid metal cannot withstand the stress transferred from the matrix, resulting in a lower Young’s modulus of composite materials. In addition, crack deflection can be effectively improved by embedding hard PDMS fiber into soft PDMS, but the Young’s modulus is still low because the reinforcement is soft PDMS^10^. In our work, the reinforcement is rigid MMT-PVA scaffold, whose original Young’s modulus reaches over 5 GPa (Supplementary Fig. 12b). Significant improvement of the Young’s modulus of our PDMS-MMT-L is due to the continuous layered PDMS-MMT scaffold designed to better withstand the applied load. At the same time, our nacre-inspired structure of PDMS-MMT-L nanocomposite exhibits improved crack resistance, leading to comparable mechanical properties of Young’s modulus and toughness, similar to natural soft materials such as skin. The comparison plot for strength and toughness is provided in Supplementary Fig. 18.

### Stiffening and toughening mechanism revealed by CFM

**Fig. 4:**
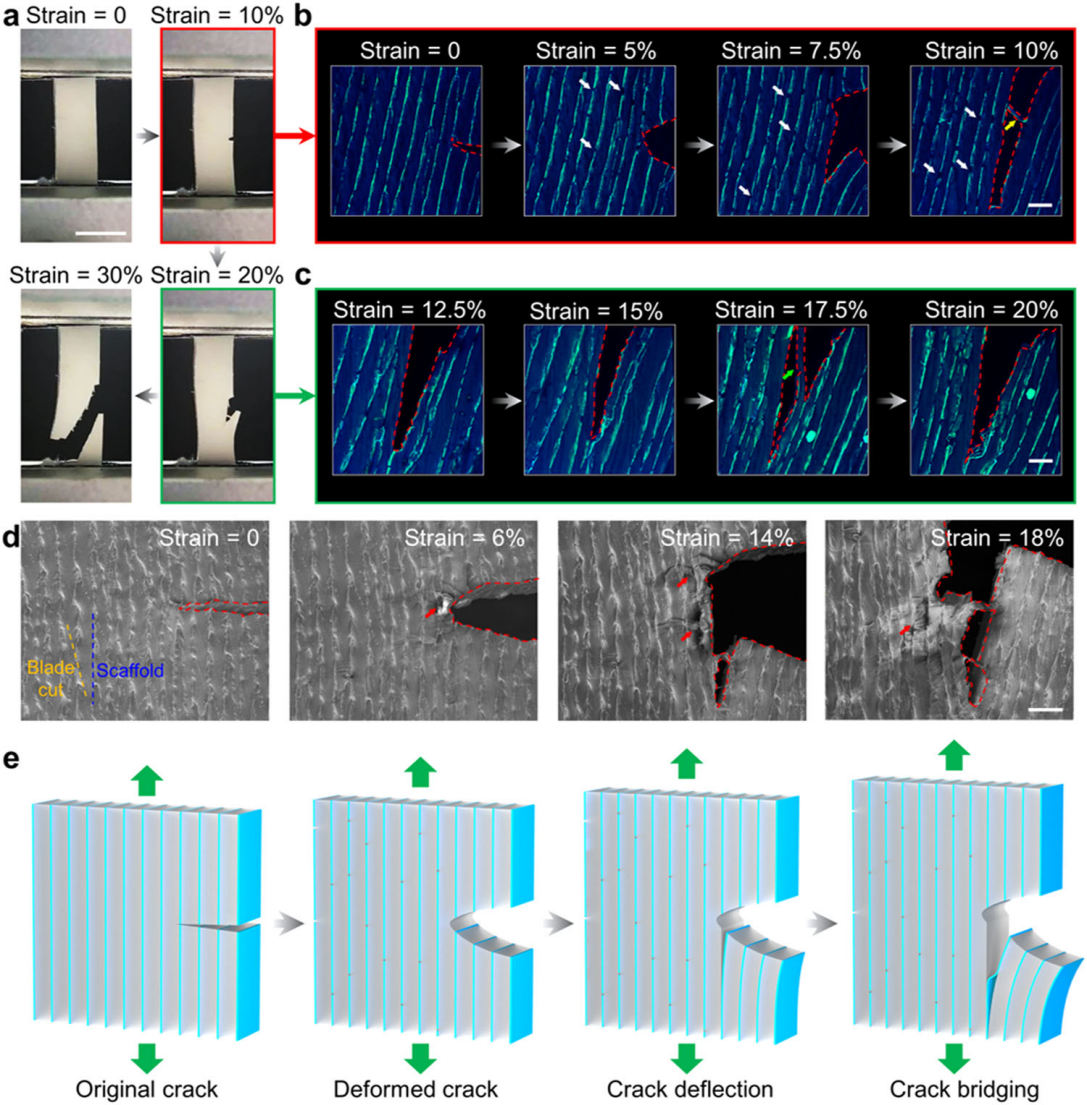
Fracture process visualized by in situ CFM. **a** Macroscale crack propagation process of PDMS-MMT-L. Scale bar: 5 mm. Microscale crack propagation of PDMS-MMT-L captured by CFM of crack initiation (**b**) and further crack propagation (**c**). Scale bar: 100 μm. **d** Microscale crack propagation of PDMS-MMT-L captured by SEM. **e** Illustration of the mechanism of crack propagation of PDMS-MMT-L. Scale bar: 100 μm.

To explore the toughening and stiffening mechanism of PDMS-MMT-L nanocomposite, we developed a method based on in situ CFM to observe the crack propagation. The fracture process is demonstrated in three steps as shown in Fig. 4a: the deformation of the crack tip, the deflection of the crack tip, and the fracture of the sample. The first two steps are the key processes to exploring the crack propagation mechanism. Thus, we focused on the crack propagation process under a small amount of stretching (Fig. 4b, c). The CFM images show that the small amount of MMT-PVA scaffold is clearly distinguished from PDMS matrix. Figure 4b shows the propagation process of a crack tip when the strain is from 0 to 10%. During this process, the crack tip starts to be stretched, resulting in deformation at the crack tip. Finite element analysis (FEA) simulation reveals that the deformation of the crack tip leads to a strong longitudinal shear stress in the PDMS matrix near the crack tip (Supplementary Fig. 19). At the same time, the rigid MMT-PVA lamellar scaffold is broken (white arrow) when the strain reaches 5%, indicating that the scaffold sustains major stress at the initial stage. Unlike traditional particle reinforcement that bears only the stress transferred from the matrix^14,16^, the continuous lamellar scaffold also directly withstands the tensile stress along the direction parallel to the layers, leading to significant improvement of the Young’s modulus. With increase of strain, the stress is more concentrated at the crack tip. When the strain reaches about 7.5%, the longitudinal shear stress makes the lamellar scaffold near the tip debond from the PDMS matrix, and initiates crack propagation. With the continued increase of longitudinal shear stress, the matrix and scaffold near the tip continuously debond with each other, leading to the subsequent crack deflection along the direction parallel to the layers. During the longitudinal crack propagation process, debonding also occurs on both sides of one layer of the lamellar scaffold, and that layer may bridge cracks, as shown by the CFM image at the strain of 10% (yellow arrow). When deflected, the crack propagated along the layered structure. As shown in Fig. 4c, when the strain is between 12.5 and 20%, the scaffold and the matrix continuously debond, leading to the longitudinal propagation of the crack. In this process, a layer of PDMS matrix may also bridge the crack (green arrow). Finally, the crack propagates to the bottom of the sample, resulting in a fracture of the sample.

To compare in situ CFM with the traditional in situ SEM characterization method, we also used in situ SEM to characterize the crack propagation process of the sample, as shown in Fig. 4d. From in situ SEM characterization, crack deflection and crack bridging could also be observed. However, the in situ SEM characterization has some problems. First, the layered structure is seriously disturbed by the surface morphology. The scratches caused by the cutting during the sample preparation process affect the observation of the layered structure. As shown in the SEM image when the strain is 0, the lamellar scaffold and the scratches are both parallel stripes but with different directions, interfering with each other. Second, scaffold and matrix are difficult to distinguish; the matrix and the small amount of scaffold are closely bonded with each other. And identifying the scaffold can rely only on the surface morphology, which shows little difference from the matrix. Therefore, the fracture of the scaffold is not clearly observed using SEM images. Finally, because the sample is not electrically conductive, it requires gold coating before SEM characterization. During the stretching process, the gold layer on the surface breaks, resulting in differences in the partial conductivity of the sample, generating the crack-like appearance (shown as red arrows). It should be noted that the CFM images in Fig. 4b, c are 3D reconstructions. The 3D images with other view angles are shown in Supplementary Fig. 20, and we especially compared the 3D CFM reconstruction image (strain: 17.5%) and the SEM image (strain: 18%) (Supplementary Fig. 21). Two interesting findings were revealed by the CFM reconstruction that could not be observed using SEM images. One is that the layered scaffold embedded into PDMS matrix was found to be fractured (yellow arrow). The fracture morphology demonstrates the process of the scaffold bearing the tension which is the key to improving the Young’s modulus. Another outcome is that the fragment of the scaffold sticking out of the PDMS ligament was clearly observed (blue arrow), showing the debonding of the scaffold from the PDMS matrix. This is why the crack is being deflected or bridged. The two salient toughening mechanisms in our layered structure, crack bridging and crack deflection, cannot be clearly observed using SEM images. Thus, the CFM reconstruction clearly and directly demonstrates the stiffening and toughening mechanisms in our layered scaffolds. In conclusion, in situ CFM can demonstrate 3D visualization, avoid the influence of surface morphology, and effectively distinguish the scaffold from the matrix, and is not limited by electrical conductivity, thus making it more effective than the traditional in situ SEM characterization methods.

Through in situ CFM characterization, we revealed the toughening mechanism of the PDMS-MMT-L nanocomposite with nacre-inspired structure (Fig. 4e). The substantial enhancement of the Young’s modulus stems from the continuous scaffold, which can bear a higher load at the beginning of the stretching process. The lamellar scaffold will first withstand the initial applied load and generate a higher tensile stress. As a result, the continuous lamellar scaffold will be broken. The improvement of toughness is due to the crack deflection and crack bridging process in the layered structure, preventing the rapid transverse propagation of the crack. The debonding of the scaffold and the matrix will continuously dissipate loading energy, similar to the extrinsic toughening mechanism of natural nacre. We have also generated 3D crack images by stacking the individual CFM slices in sequence, following the technique described by Podsiadlo et al.^47^, which can be seen in Supplementary Fig. 22. We compared the crack growth process of pure PDMS and PDMS-MMT-R nanocomposite. Their cracks both grow instantaneously, and the crack propagation processes are even faster than the minimum time step of in situ CFM and SEM characterization (Supplementary Figs. 23 and 24). The FEA also reveals that the debonding of the scaffold and the matrix is a significant contributor to crack initiation and deflection (Supplementary Fig. 19), confirming the toughening mechanism we proposed.

## Discussion

In summary, we have prepared a nacre-inspired layered PDMS-MMT nanocomposite. Its Young’s modulus and toughness have been significantly improved to 23 and 12 times higher than those of pure PDMS, respectively. The mechanical properties of the PDMS-MMT-L nanocomposite are comparable to natural soft materials such as skin or cartilage. At the same time, we introduced AIE molecules into PDMS-MMT-L nanocomposite, and successfully realized the 3D reconstruction of the microstructure and the in situ characterization of the fracture process by CFM. The characterization based on CFM overcomes the disadvantages of traditional SEM characterization, including interference from surface morphology, difficulty in distinguishing different components, false appearance by gold coating, and the obstacle of 3D reconstruction. Thus, the toughening mechanism of nacre-inspired layered PDMS-MMT nanocomposite was revealed by the AIE-assisted CFM characterization, which provides an avenue for exploring the toughening and stiffening mechanism of nanocomposites.

## Methods

### Materials

PVA (Mw 13000~23000, 87–89%hydrolyzed) was purchased from Aldrich. MMT was purchased from Nanocor. PDMS (Sylgard 184 silicone elastomer) was purchased from Dow Corning. The p-toluenesulfonic acid monohydrate was purchased from Adamas. The 1-pyrenamine was purchased from the Aldrich. TPE-CHO was synthesized by Professor Ben Zhong Tang’s group.

### Exfoliation of MMT nanosheets

The natural MMT powder (3 g) was dispersed into 500 mL deionized water and stirred for a week. Then, centrifugation was conducted with a speed of 541 g for 10 min for three times to remove unexfoliated MMT. A resultant homogeneous MMT suspension was obtained followed by evaporation to generate the solid MMT nanosheets.

### Fabrication of PVA-TPE

The PVA powder (1 g) was dissolved into 10 mL dimethyl sulfoxide at 90 °C for 20 min to obtain transparent PVA solution. Then, 10 g TPE-CHO powder and 130 mg p-toluenesulfonic acid were added to the PVA solution after cooling under continuous stirring. The mixed solution was stirred at 80 °C for 4 h and the yellow solution gradually turned colorless. Then, 500 mL acetone was poured into the resultant reaction mixture to precipitate the PVA-TPE. The white flocculent precipitation was filtrated and washed by acetone three times. The as-prepared PVA-TPE was dried at 45 °C to remove residual acetone.

### Fabrication of MMT-PVA scaffold

The PVA-TPE was dissolved in deionized water at 80 °C and stirred for 10 min to yield a transparent PVA-TPE solution (50 mg·mL^−1^). The solid MMT nanosheets were also dispersed in deionized water and stirred for 12 h to obtain a viscous MMT suspension (50 mg·mL^−1^). The PVA-TPE and MMT nanosheets were mixed with a weight ratio of 1:1. Then the MMT-PVA lamellar scaffolds (15 × 10 × 10 mm^3^) were prepared by bidirectional freeze-casting. After freeze-drying in a vacuum freeze-dryer (<1 Pa) for 2 days, the ice was removed, leaving a lamellar scaffold.

### Preparation of nacre-inspired layered PDMS-MMT-L nanocomposite

The PDMS-MMT-L nanocomposite was fabricated via infiltrating the PDMS (the ratio of base to curing agent was 5:1) into an MMT-PVA scaffold, followed by a curing process at 90 °C for 12 h. To eliminate the void between the PDMS and scaffold, the infiltration process was helped by vacuum. For comparison, the PDMS-MMT-R was fabricated as follows. The mixed solution containing PVA-TPE and MMT nanosheets with a weight ratio of 1:1 was poured into tetrahydrofuran liquid to precipitate the mixture of PVA-TPE and MMT. Centrifugation at 2000 rpm for 10 min was applied to separate the precipitation and the resultant precipitation was washed by tetrahydrofuran. The as-prepared PVA-MMT mixture was dispersed into tetrahydrofuran via sufficient sonification. Then, the dispersion of PVA-MMT was mixed with PDMS base followed by evaporation of tetrahydrofuran at room temperature. The curing agent was added into the PDMS base mixed with MMT-PVA and cured at 90 °C for 12 h. For both the PDMS-MMT-L and PDMS-MMT-R, the 1-aminopyrene was added into the PDMS matrix before curing for the subsequent CFM characterization. By changing the weight ratio of MMT to PVA, the PDMS-MMT-I and PDMS-MMT-II nanocomposites were obtained with a weight ratio of 1:3 and 3:1, respectively. The nanocomposites with different interlayered spacing were fabricated by using different cold substrates, including copper, stainless steel, and cast iron. The PDMS-MMT-L nanocomposite was obtained from cast iron with an interlayer spacing of 30–50 μm. The PDMS-MMT-III nanocomposite was fabricated from the copper substrate with a higher thermal conductivity, resulting in an interlayer spacing of 15–40 μm. The steel substrate with a lower thermal conductivity led to a larger interlayer spacing of 70–160 μm in the PDMS-MMT-IV nanocomposite.

### Finite element method analysis

A 2D nonlinear finite element model under plane stress condition was constructed by the software ABAQUS v6.12. The model was developed as a square PDMS (600 × 600 μm^2^) with embedded MMT-PVA layers. The interlayer spacing was 50 μm and the thicknesses of MMT-PVA layers were randomly from 1 μm to 4 μm. An experiment stress-strain data and a Poisson ratio of 0.47 were used as the constitutive relation of PDMS according to the Marlow model for hyperelastic material. The Young’s modulus for MMT-PVA layers was 5 GPa with a Poisson ratio of 0.3. A cohesive interaction was used to simulate the bonding between PDMS matrix and MMT-PVA layers. The debonding stress was assumed as 100 kPa for both tensile and shear modes. We applied the extended finite element method to simulate the break of MMT-PVA layers. Given that massive defects exist in these MMT-PVA layers, some crack propagating regions were arranged on the lamellar scaffold. The damage stress was set as 120 MPa, and an energy-based Benzeggagh-Kenane (BK) damage evolution criterion was used with an energy release rate of 2 N·m^−1^. The loads were applied as a fixed location of bottom edge and a upside displacement of top edge.

### Characterization

The microstructures of the scaffolds and nanocomposites were observed using a HITACHI-S8010 at an accelerating voltage of 5.0 kV and current of 10 μA. The samples were sputtered with platinum before SEM characterization. TGA was conducted on a Netzsch STA449F3 from 40 to 800 °C with a heating rate of 10 °C·min^−1^ under air atmosphere. Fluorescence microscope images were obtained from a confocal laser scanning microscope system (Leica, A1). For the lamellar scaffold, the CFM images were obtained with a 405-nm laser. For PDMS-MMT-L and PDMS-MMT-R, the CFM images were taken under a two-channel mode. The green channel was set with an excitation wavelength of 488 nm and an emission wavelength of 500–550 nm while the blue channel was set with an excitation wavelength of 405 nm and an emission wavelength of 417–477 nm. The FTIR was conducted on a Nicolet (iN10MX) and the AFM image was obtained from a Bruker Multimode 8.

The mechanical properties were tested as follows. The sample was cut into a stripe with a length of 5 mm, a width of 3 mm, and a thickness of about 0.3 mm. The lamellar direction was along the longitudinal direction of the sample. Both ends of the sample were fixed on sandpaper to avoid slippage between the sample and clamps. The stress-strain curves were obtained by a SUNS UTM4103 Tester with a loading rate of 0.1 mm·min^−1^. Toughness was characterized by the fracture energy (*Γ*), which was calculated according to a conventional reported method^10,14,63^. The tensile tests were conducted on notched samples and compared with unnotched samples to calculate the fracture energy. A force-displacement curve was measured for both the notched and unnotched samples with the same size. The critical displacement (*L*_c_) was measured for the notched sample where the notch turns into a fast-advancing crack. The corresponding unnotched sample was tested to obtain the mechanical work at the *L*_c_ (U(*L*_c_)). The final fracture energy was calculated as *Γ* = U(*L*_c_)/*A*, where the *A* is the cross-sectional area of the samples.

## Supplementary information

Supplementary Information

## Data Availability

The data that support the findings of this study are available from the corresponding authors upon reasonable request.
