# Supplementary material for: Stiff and tough PDMS-MMT layered nanocomposites visualized by AIE luminogens
Source: Nat Commun. 2021 Jul 27;12:4539. doi: 10.1038/s41467-021-24835-w (PMC8316440; doi:10.1038/s41467-021-24835-w)
Supplement: Supplementary file 1 — Supplementary Information [file 41467_2021_24835_MOESM1_ESM.pdf]

Supplementary Information

Stiff and Tough PDMS-MMT Layered Nanocomposites Visualized by AIE Luminogens

Jingsong Peng[1], Antoni P. Tomsia[1], Lei Jiang[1], Ben Zhong Tang[2]*, and Qunfeng Cheng[1,3]*

[1]School of Chemistry, Key Laboratory of Bio-inspired Smart Interfacial Science and Technology of Ministry of Education, Beijing Advanced Innovation Center for Biomedical Engineering, Beihang University, Beijing 100191, China.

[2]Department of Chemistry, The Hong Kong Branch of Chinese National Engineering Research Center for Tissue Restoration and Reconstruction, Institute of Molecular Functional Materials, Division of Life Science and State Key Laboratory of Molecular Neuroscience, The Hong Kong University of Science and Technology, Clear Water Bay, Kowloon, Hong Kong 999077, China.

[3]School of Materials Science and Engineering, Zhengzhou University, Zhengzhou 450001, China

*Correspondence should be addressed to Ben Zhong Tang and Qunfeng Cheng, Email: tangbenz@ust.hk and cheng@buaa.edu.cn

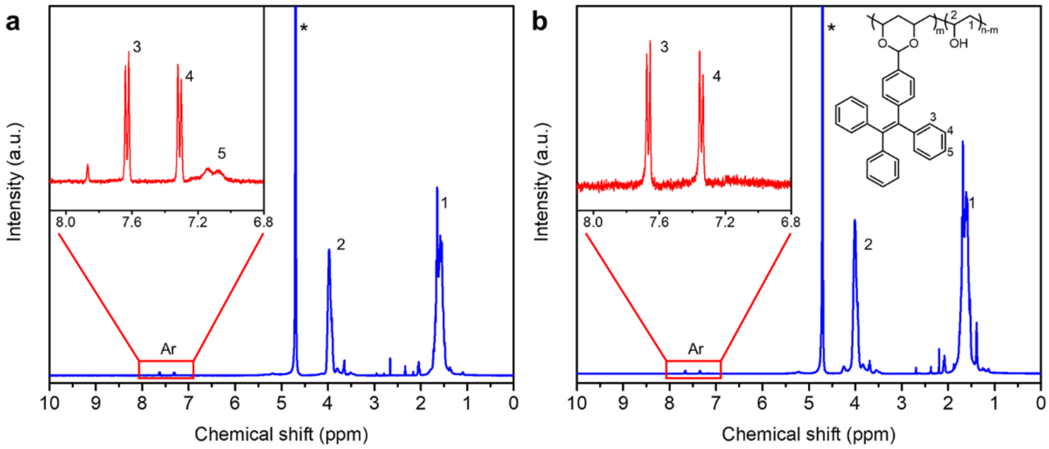

Supplementary Figure 1. ^{1}H NMR spectrum of PVA-TPE with a raw material ratio of TPE-CHO to PVA of 1:100 (**a**) and 1:50 (**b**) in D_2O. The solvent peak is marked with an asterisk.

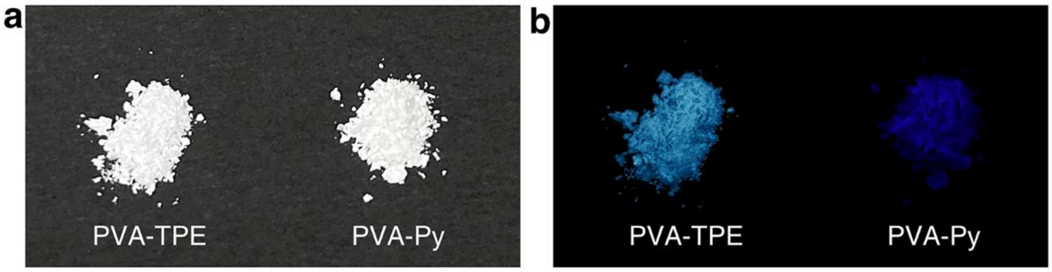

Supplementary Figure 2. Digital photos of PVA-TPE and PVA-Py powders under room light (**a**) and 365 nm UV light (**b**).

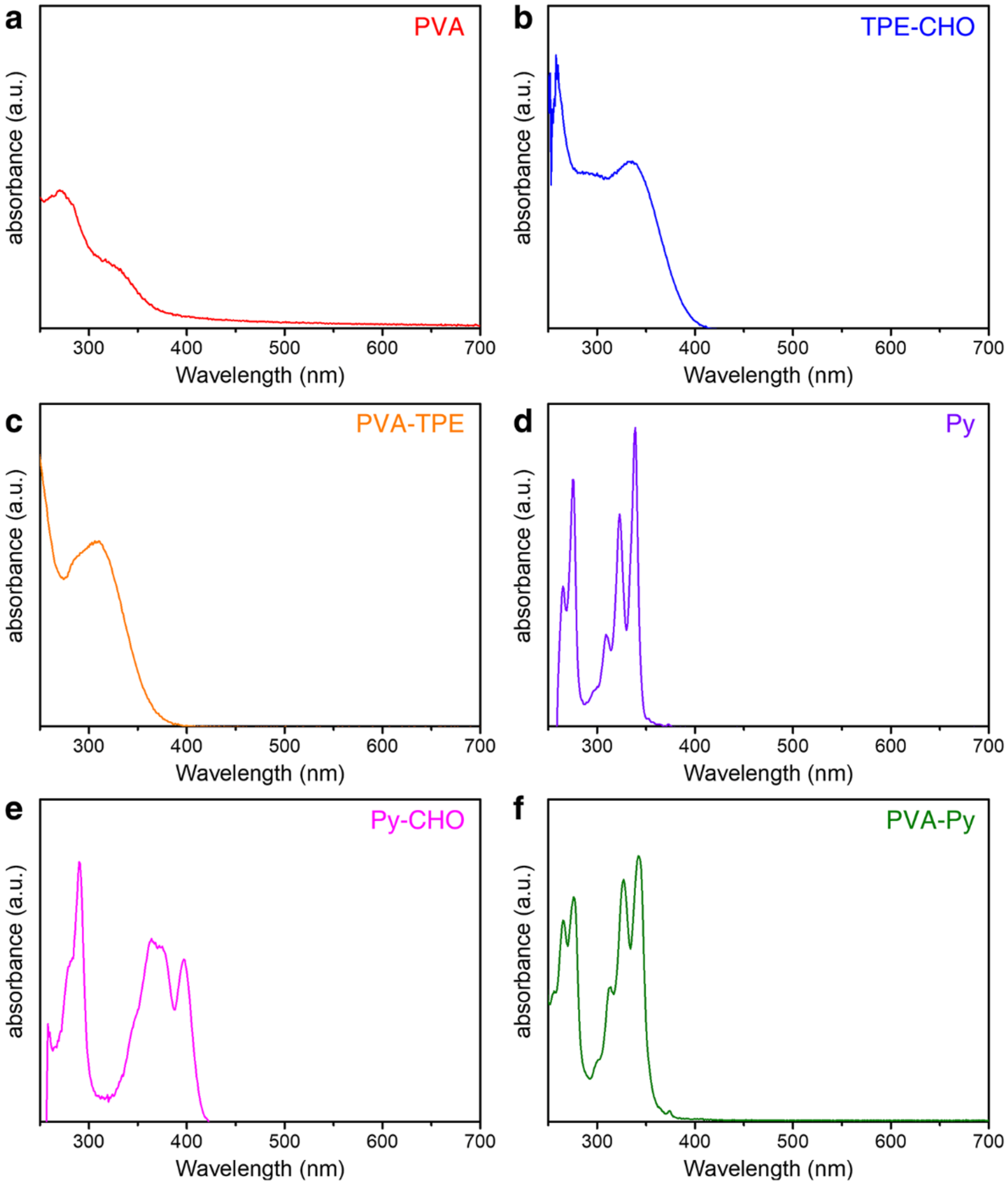

Supplementary Figure 3. The UV-vis absorption spectra of (**a**) PVA, (**b**) TPE-CHO, (**c**) PVA-TPE, (**d**) Py, (**e**) Py-CHO, and (**f**) PVA-Py.

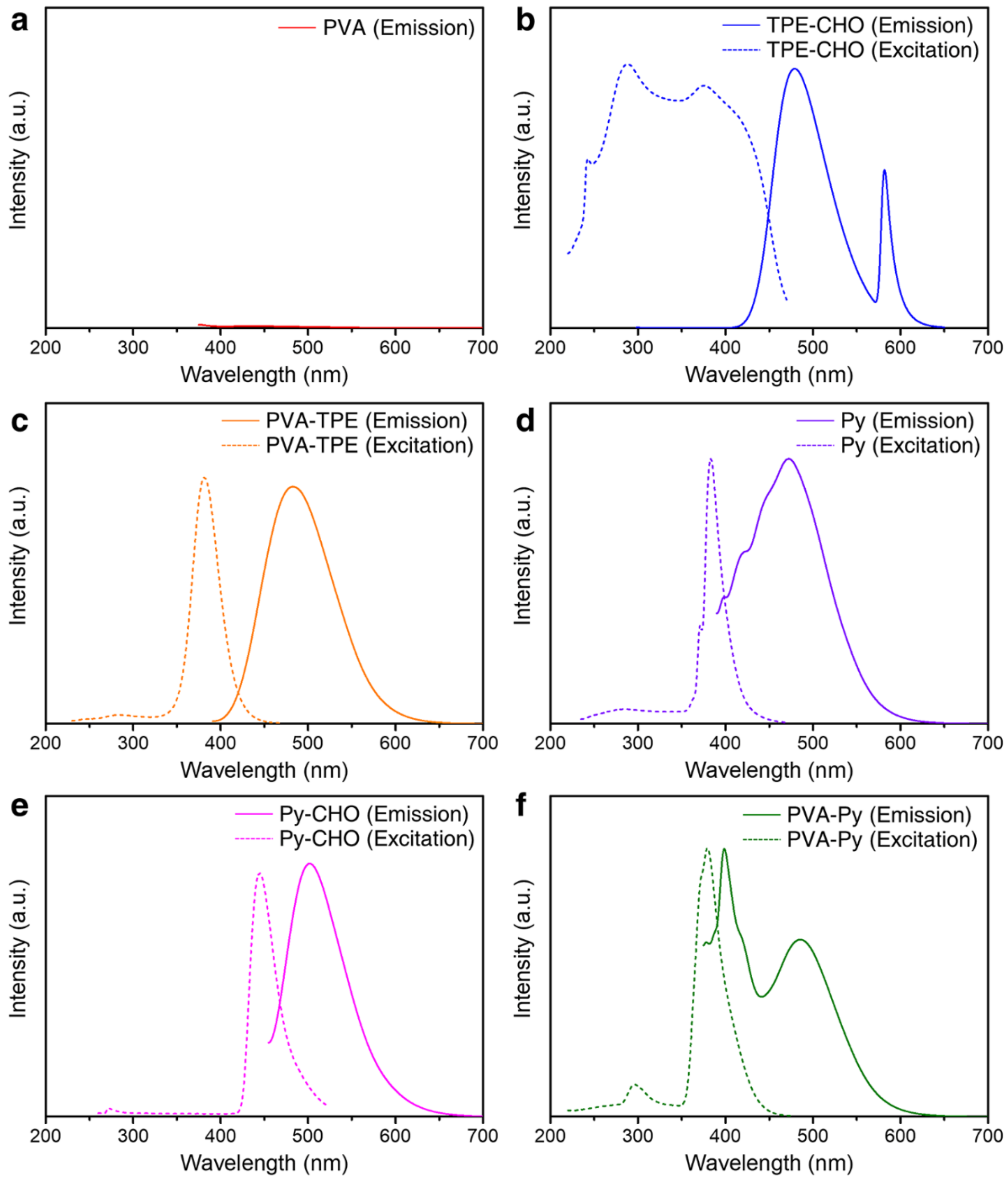

Supplementary Figure 4. The fluorescence spectra of (**a**) PVA, (**b**) TPE-CHO, (**c**) PVA-TPE, (**d**) Py, (**e**) Py-CHO, and (**f**) PVA-Py. There is no overlap between the absorption spectrum of Py and the emission spectrum of PVA-TPE or between the emission spectrum of Py and the absorption spectrum of PVA-TPE. Thus, the fluorescence resonance energy transfer cannot occur between Py and PVA-TPE.

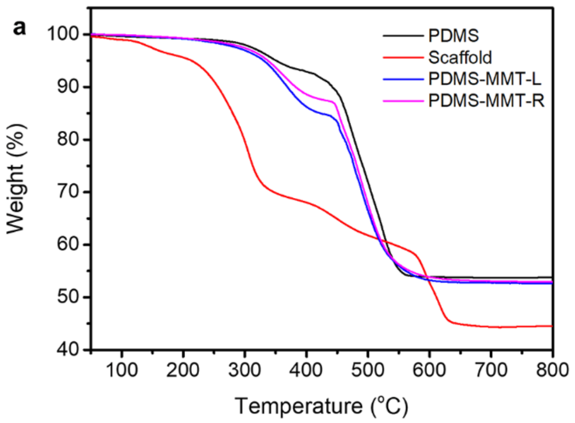

b

Sample	Weight loss (800 °C, %)	PDMS Content (wt%)
PDMS	46.21	100
PDMS-MMT-L	47.03	91.2
PDMS-MMT-R	47.36	87.7
Scaffold	55.49	0

Supplementary Figure 5. (**a)** TGA curves of PDMS, lamellar scaffold, PDMS-MMT-L, and PDMS-MMT-R nanocomposites. The TGA tests were conducted under air atmosphere with a heating rate of 10 °C·min^{-1}. (**b)** The exact PDMS content of PDMS-MMT-L and PDMS-MMT-R nanocomposites determined by TGA.

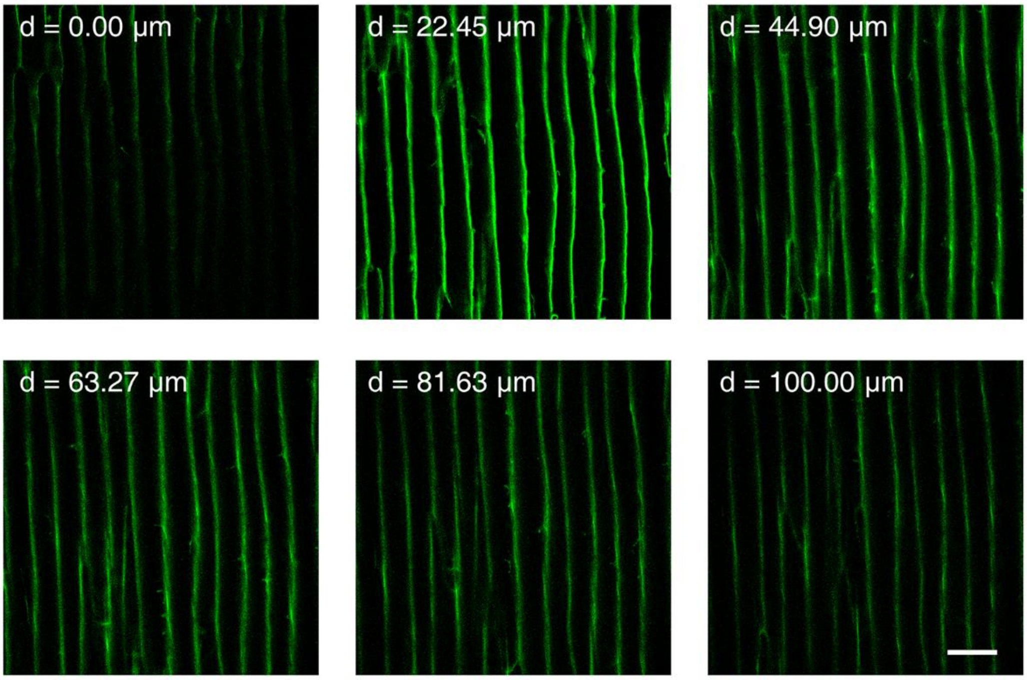

Supplementary Figure 6. CFM images of the lamellar scaffold under different depths. The images show different cross sections of the lamellar scaffold at different depths, showing the microstructure of the lamellar scaffold under the surface. The bridges between the layers and the edges on the layers can be well distinguished. Scale bar: 100 μm.

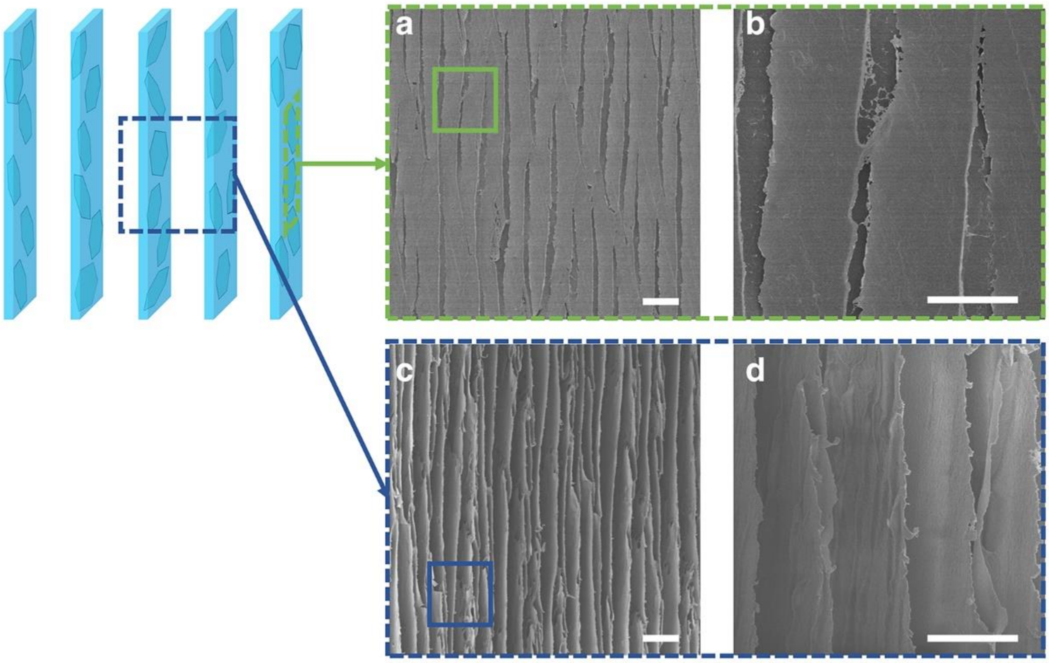

Supplementary Figure 7. SEM images of a lamellar scaffold on the surface of the layers (**a,b**) and cross section of the layers (**c,d**). Due to the limits of the SEM characterization, the 3D information of the microstructure of a lamellar scaffold can be obtained only by preparing different samples. Furthermore, the sample preparation process, including cutting or peeling of these MMT-PVA layers, may break microstructures, such as bridges between layers or edges on the layers. Therefore, the characterization results from SEM may not demonstrate the original microstructure of the lamellar scaffold. Scale bar for (**a**) and (**c**): 100 μm. Scale bar for (**b**) and (**d**): 50 μm.

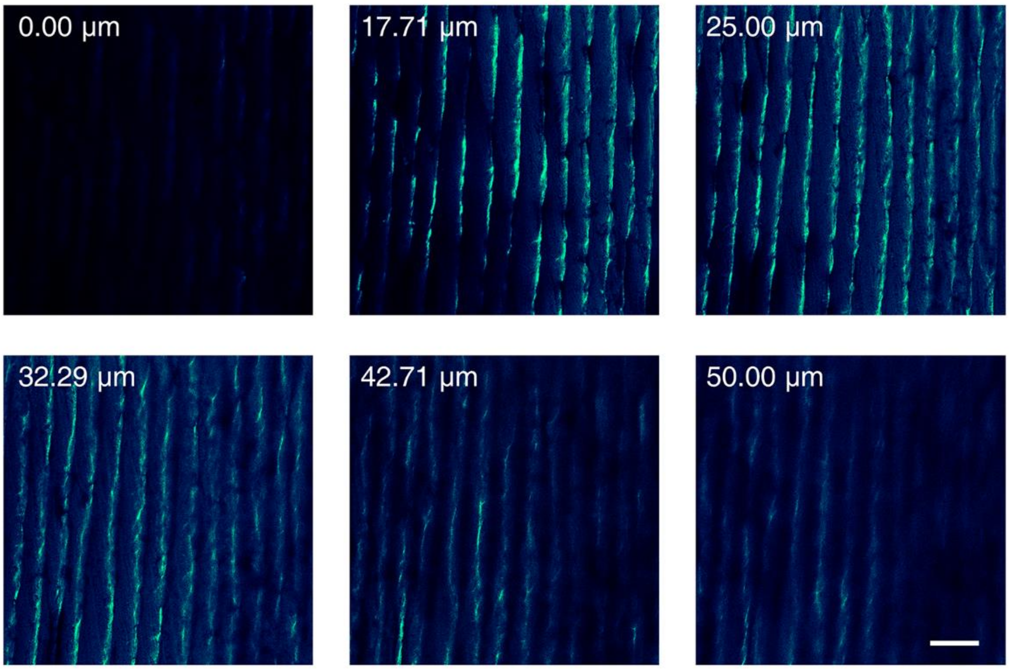

Supplementary Figure 8. CFM images of PDMS-MMT-L under different depths. These images prove that the lamellar scaffold is tightly bonded with the PDMS matrix without voids. CFM can also avoid the interference from the surface. For example, at the depth of 17.71 μm, the CFM image shows that the surface is uneven due to how it was cut during the sample preparation. While the depth is 25.00 μm, the CFM image demonstrates a uniform layered structure including scaffold and matrix. Scale bar: 100 μm.

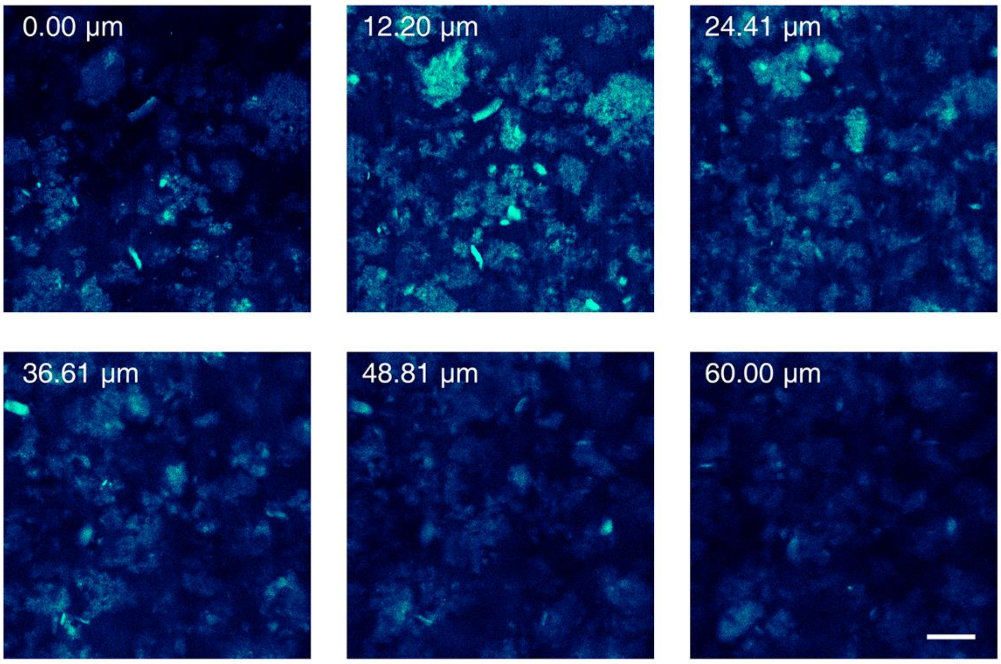

Supplementary Figure 9. CFM images of PDMS-MMT-R under different depths. These images show the different distribution of MMT-PVA particles at different depths. Scale bar: 100 µm.

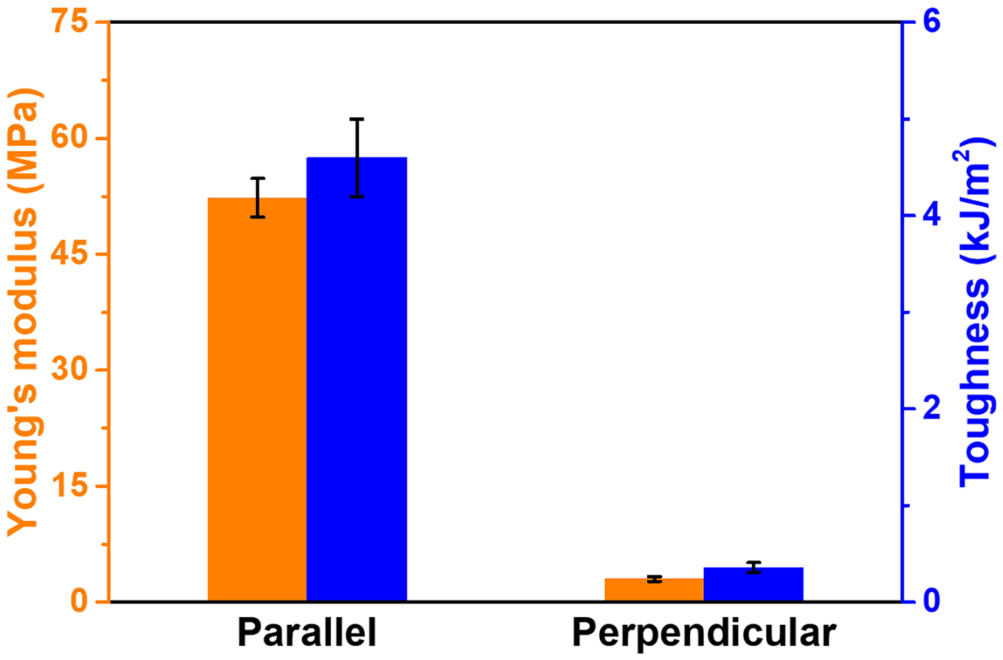

Supplementary Figure 10. Comparison of Young's modulus and toughness between PDMS-MMT-L nanocomposites under tensile stress that is parallel to the layered scaffold and perpendicular to it. The resultant anisotropies for the Young's modulus and toughness are 8.6 and 41.8, respectively. For all panels, error bars are mean ± SD.

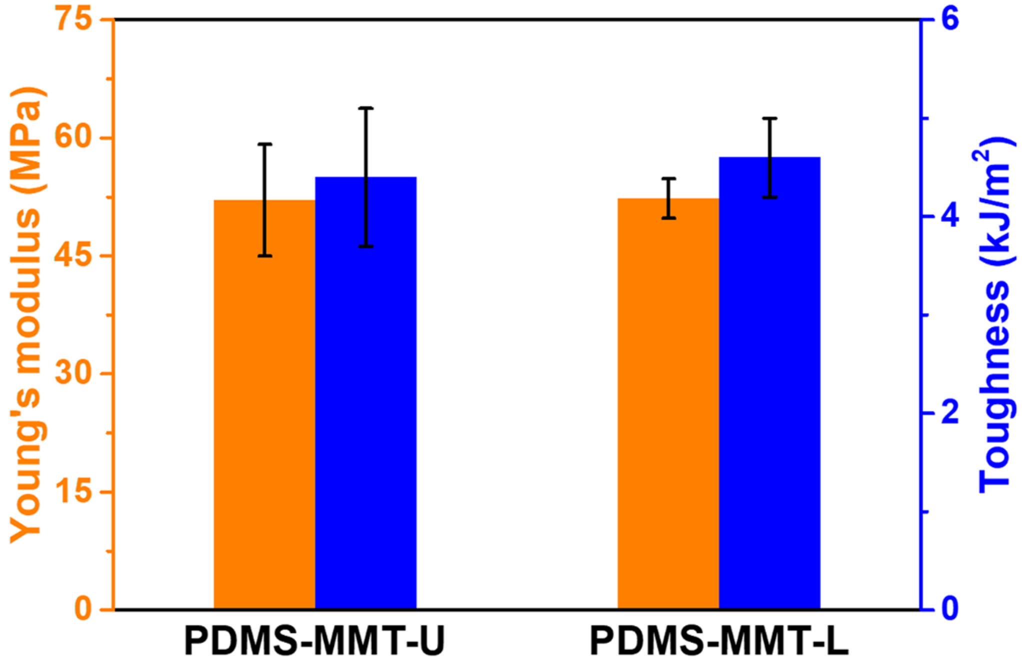

Supplementary Figure 11. Comparison of Young's modulus and toughness between PDMS-MMT-U and PDMS-MMT-L nanocomposites. The unlabeled PDMS-MMT-U nanocomposite demonstrates a Young's modulus of 52.1 ± 7.1 MPa and a toughness of 4.4 ± 0.7 kJ/m^3. For all panels, error bars are mean ± SD.

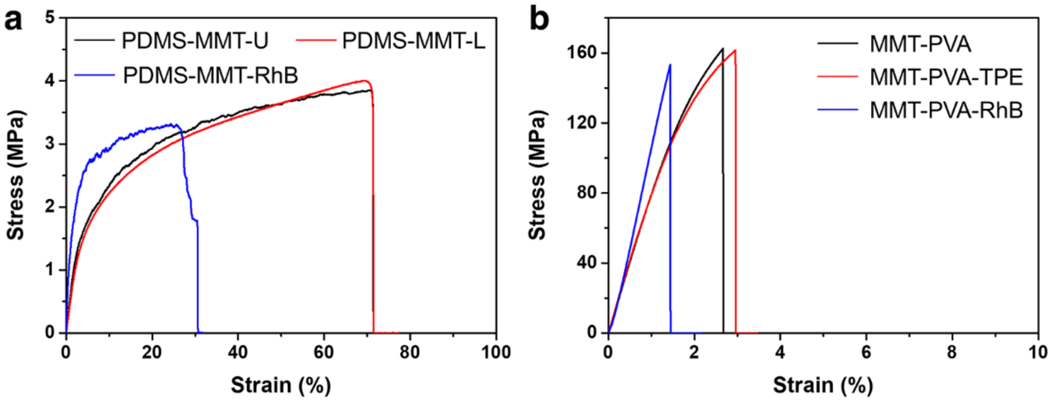

Supplementary Figure 12. (**a**) Stress-strain curves of PDMS-MMT-U, PDMS-MMT-L, and PDMS-MMT-RhB nanocomposites. With the addition of RhB, the Young's modulus increases to 160.5 ± 15.7 MPa while the strain drops to about 30%, demonstrating a more brittle nanocomposite. (**b**) Stress-strain curves of MMT-PVA, MMT-PVA-TPE, and MMT-PVA-RhB films. The Young's modulus of MMT-PVA, MMT-PVA-TPE, and MMT-PVA-RhB films is 8.1 ± 0.1 GPa, 7.9 ± 0.4 GPa, and 11.9 ± 0.6 GPa, respectively. Thus, the addition of RhB also embrittles the MMT-PVA-RhB film, indicating that RhB significantly influences the mechanical properties of MMT-PVA scaffold.

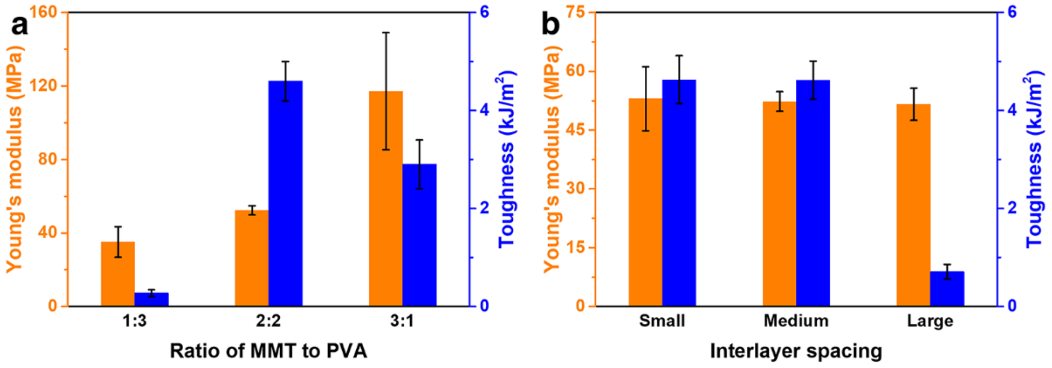

Supplementary Figure 13. Comparison of Young's modulus and toughness between different nanocomposites with different ratios of MMT to PVA (**a**) and interlayer spacing (**b**). For all panels, error bars are mean ± SD.

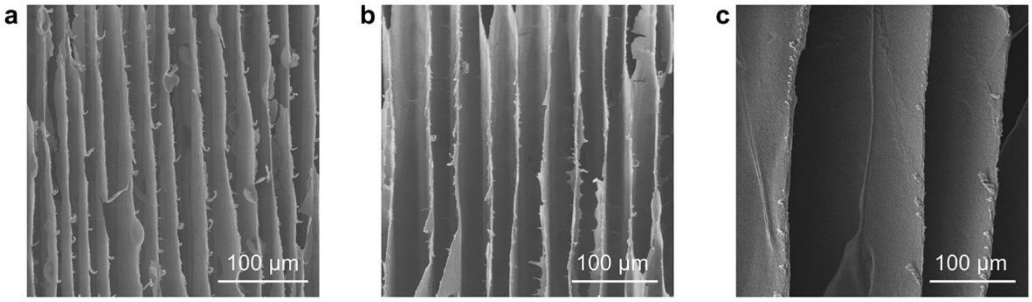

Supplementary Figure 14. SEM images of layered scaffolds with interlayer spacing of 15~40 μm (**a**), 30~50 μm (**b**), and 70~160 μm (**c**). The different interlayer spacings are achieved by controlling the freezing rate via using cold substrates with different materials including copper, stainless steel, and cast iron.

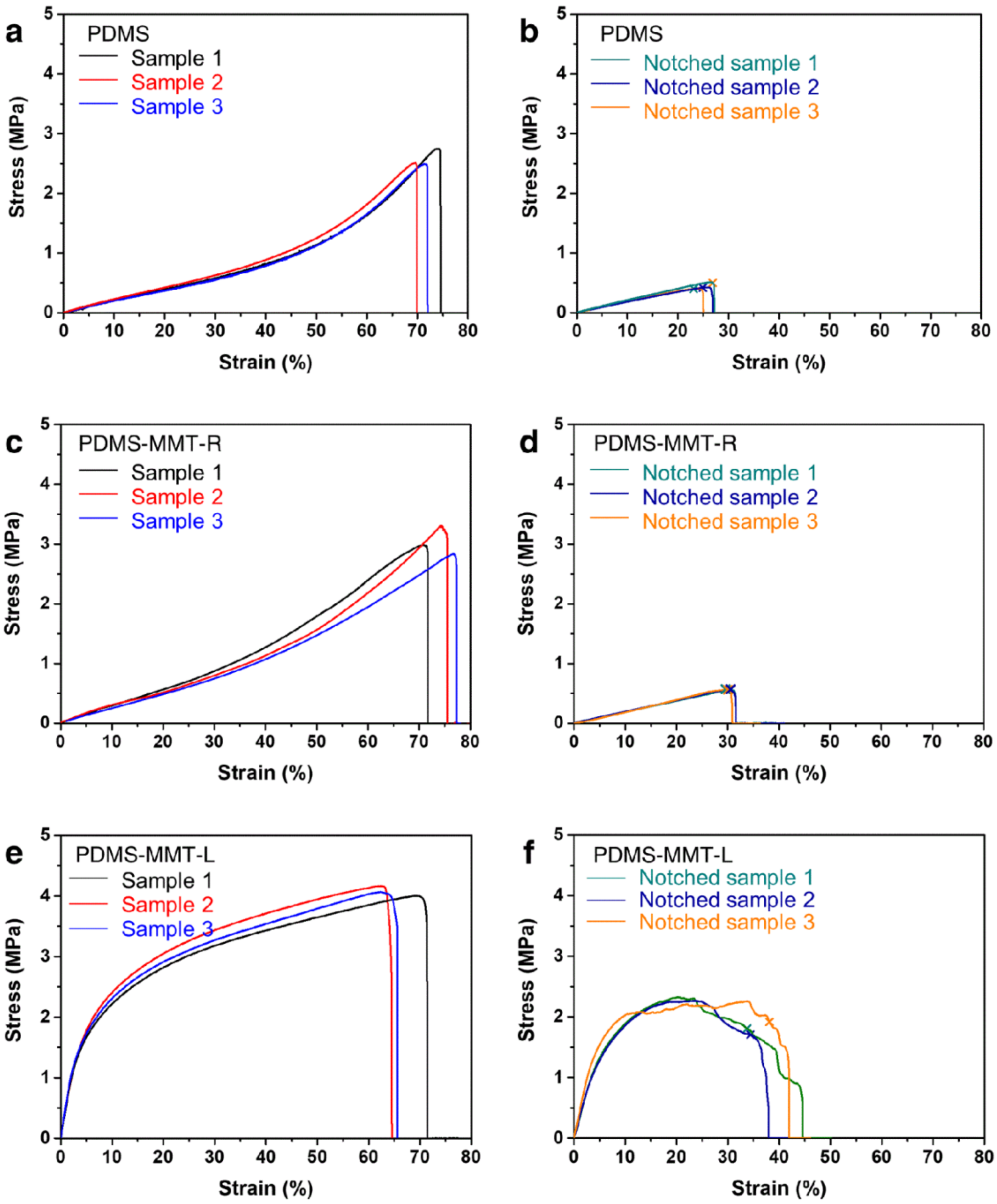

Supplementary Figure 15. Stress-strain curves of PDMS (**a**) notched PDMS (**b**) PDMS-MMT-L (**c**) notched PDMS-MMT-L (**d**) PDMS-MMT-R (**e**) and notched PDMS-MMT-R (**f**). These curves all show good repeatability.

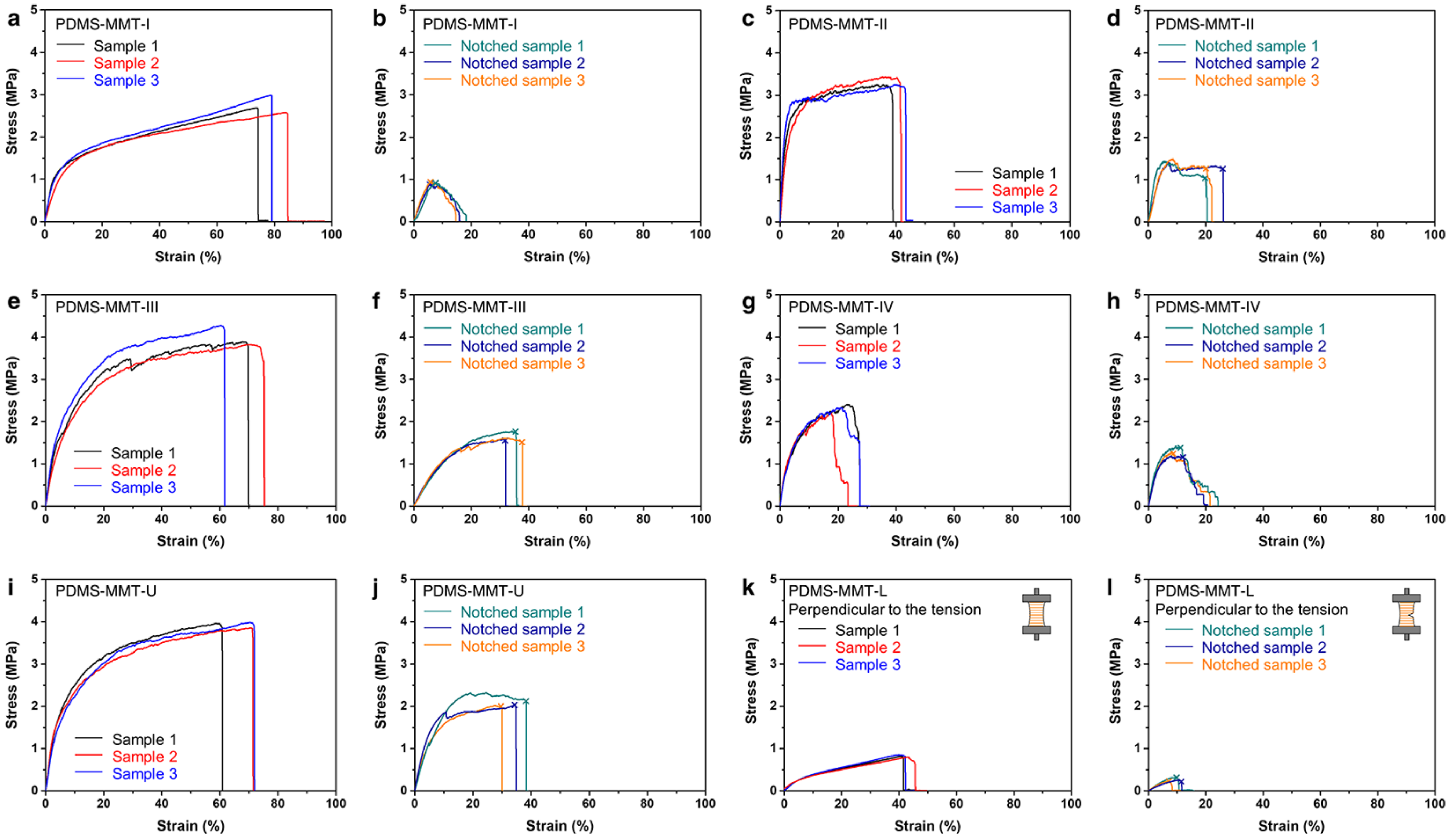

Supplementary Figure 16. Stress-strain curves of PDMS-MMT-I (**a**) notched PDMS-MMT-I (**b**) PDMS-MMT-II (**c**), notched PDMS-MMT-II (**d**), PDMS-MMT-III (**e**) notched PDMS-MMT-III (**f**) PDMS-MMT-IV (**g**) notched PDMS-MMT-IV (**h**) PDMS-MMT-U (**i**) notched PDMS-MMT-U (**j**) PDMS-MMT-L (perpendicular) (**k**) and notched PDMS-MMT-L (perpendicular) (**l**).

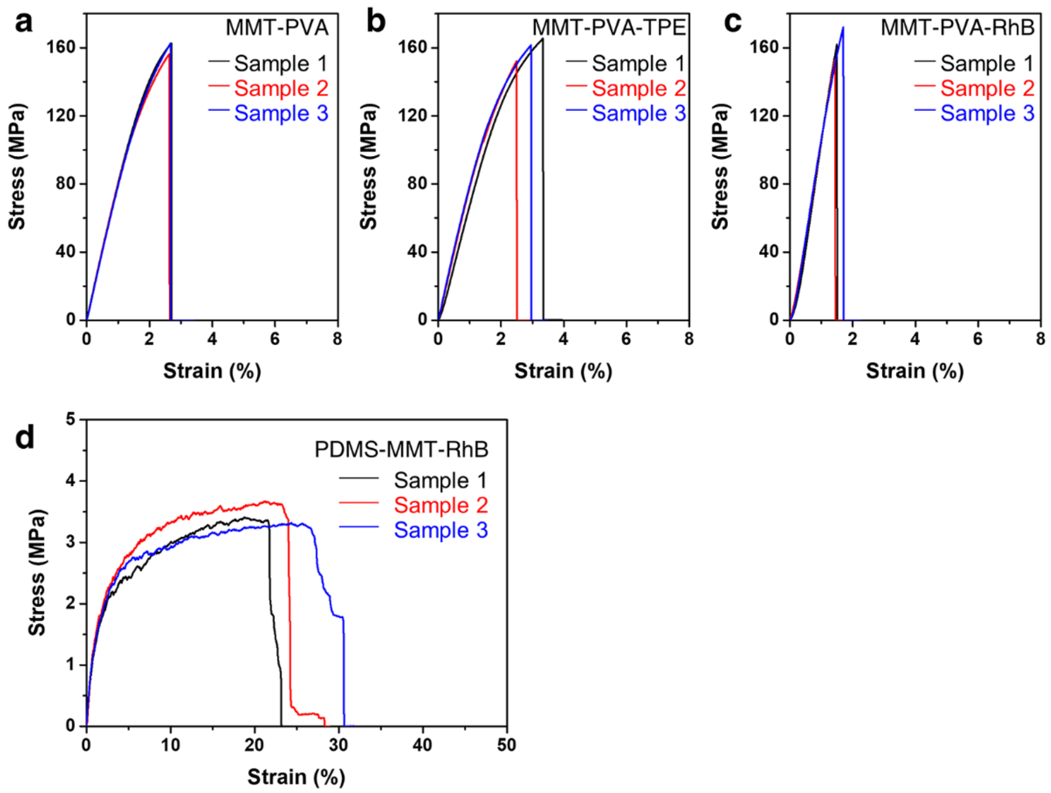

Supplementary Figure 17. Stress-strain curves of MMT-PVA (**a**) MMT-PVA-TPE (**b**) and MMT-PVA-RhB (**c**) films and PDMS-MMT-RhB nanocomposite (**d**).

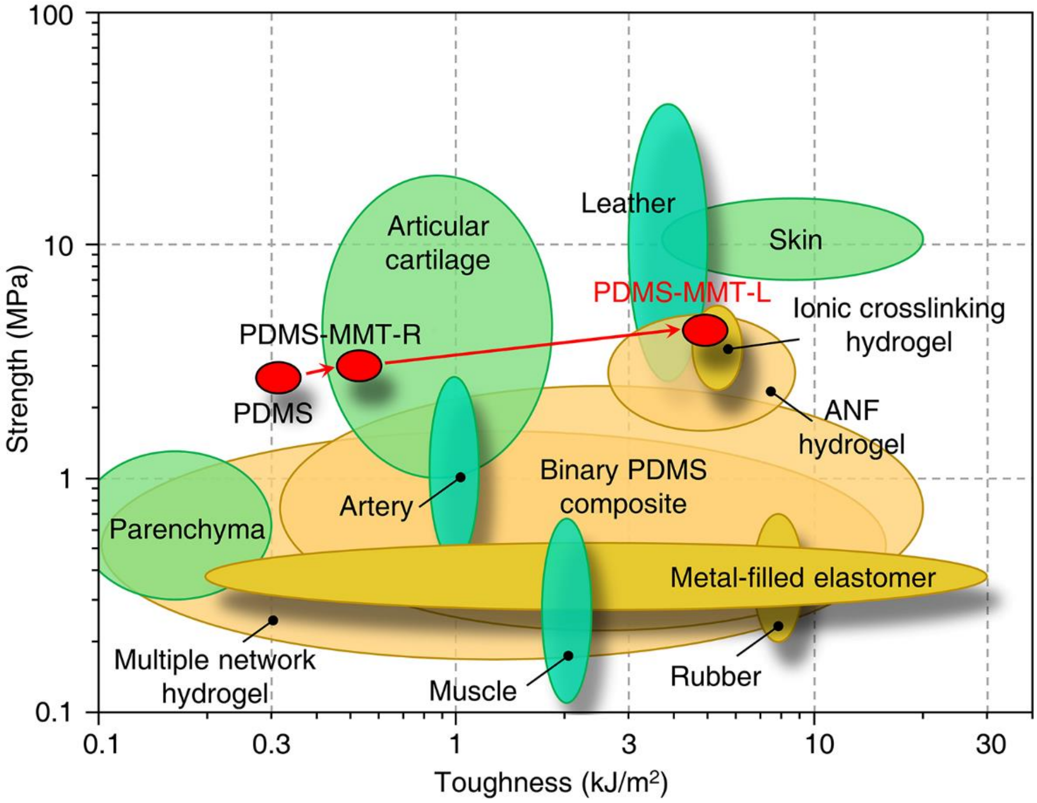

Supplementary Figure 18. Comparison of toughness and strength between pure PDMS, PDMS-MMT-R, and PDMS-MMT-L nanocomposite, and some artificial and natural soft materials. The PDMS-MMT-L boosts the mechanical properties to a level comparable to natural materials.

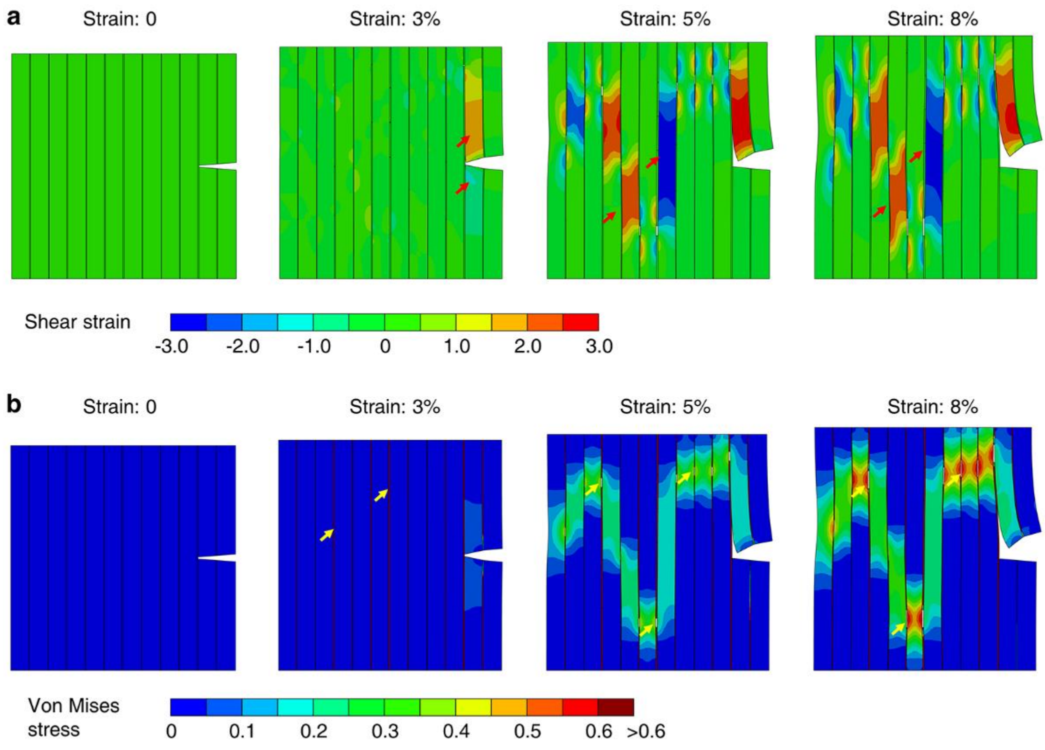

Supplementary Figure 19. FEA results of shear strain (**a**) and von Mises stress (**b**) tests of nacre-inspired PDMS-MMT-L nanocomposite. The result of the shear strain test demonstrates that when the crack tip is deformed (longitudinal strain: 3%), the PDMS matrix will generate a concentrated shear strain (the red arrows), leading to a shear stress which will push the matrix to be separated from the layer generating crack deflection. Furthermore, on the region that is far from the crack tip, the matrix will also generate local shear strain due to increased stretching of the sample (longitudinal strain: 5% and 8%, the red arrows). These regions can be the possible location of crack bridging. The results of von Mises stress testing reveal that the lamellar scaffold will first bear the load to generate a significantly higher stress than the matrix shown as (longitudinal strain: 3%, the yellow arrows). With the increase of longitudinal strain, the scaffold will first break to generate voids (longitudinal strain: 5% and 8, the yellow arrows).

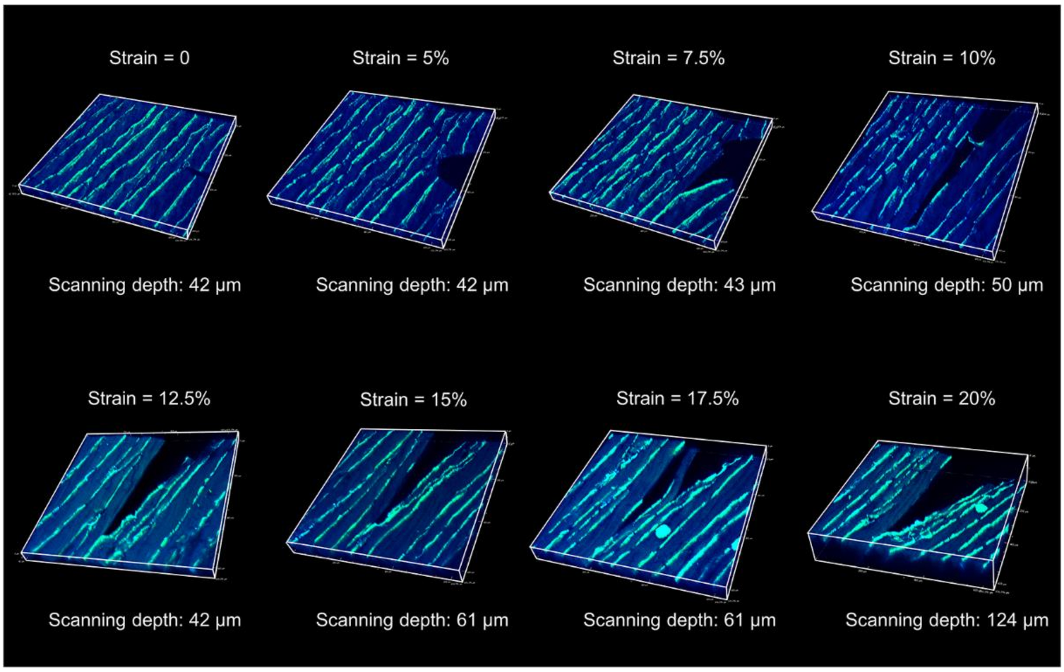

Supplementary Figure 20. 3D reconstruction of microscale crack propagation of PDMS-MMT-L captured by CFM.

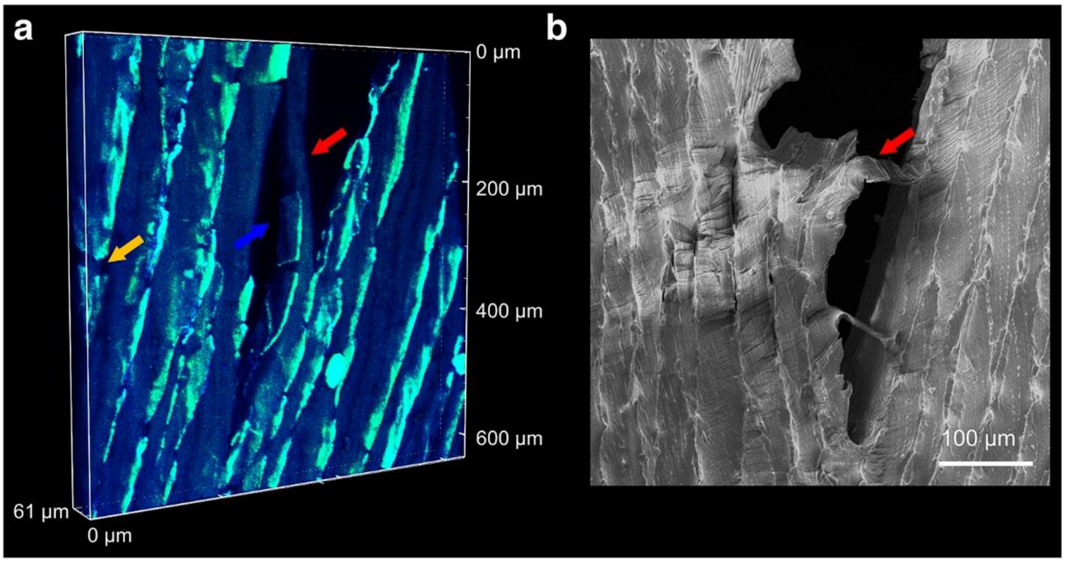

Supplementary Figure 21. Comparison between a 3D CFM reconstruction (**a**) and a SEM image (**b**) of crack propagation. The red arrows point out the ligaments caused by crack bridging at the 3D CFM reconstruction and 2D SEM image. From the 3D CFM construction, the cracked scaffold residues are found to stick on the PDMS layer (blue arrow) and the cracked scaffold embedded into the PDMS matrix is also observed (yellow arrow). The SEM image, however, cannot reveal these morphologies and is obstructed by the uneven gold coating and surface morphology during the tension process.

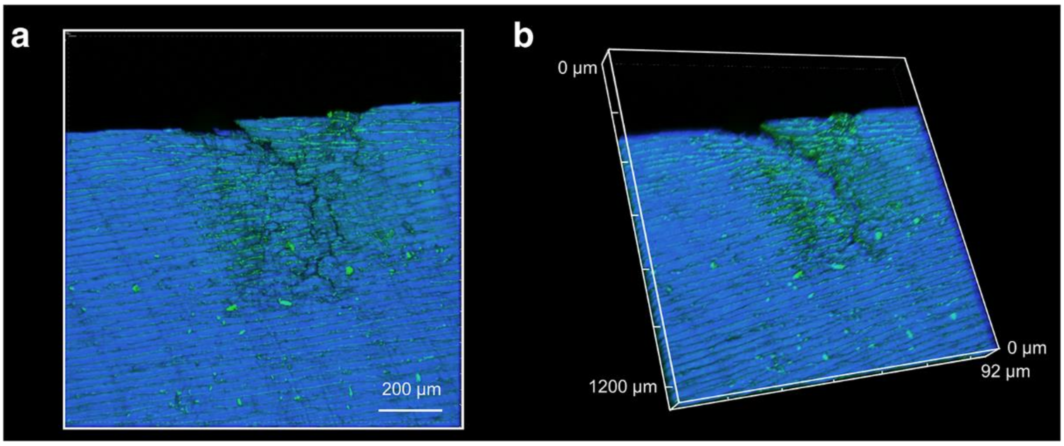

Supplementary Figure 22. Front view (**a**) and 3D view (**b**) of a 3D CFM reconstruction of the crack caused by a pressed steel ball. To generate the crack, a steel ball has been pressed into the PDMS-MMT-L nanocomposite using a hydraulic press. The resultant crack also shows a trajectory with abundant crack deflection.

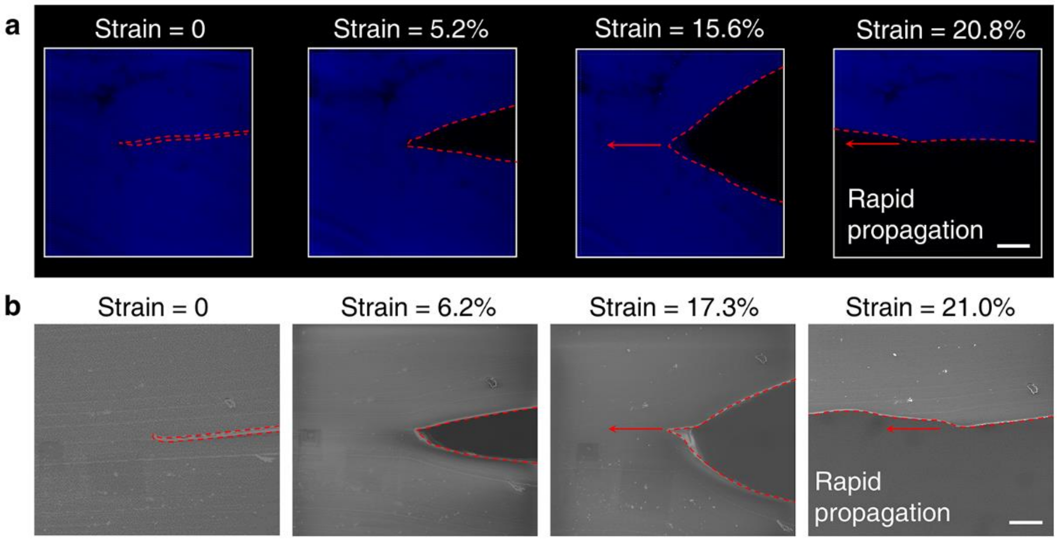

Supplementary Figure 23. In situ CFM characterization (**a**) and SEM characterization (**b**) of crack propagation of pure PDMS. Both the CFM and SEM characterization show a rigid unstable crack propagation. Scale bar: 100 μm.

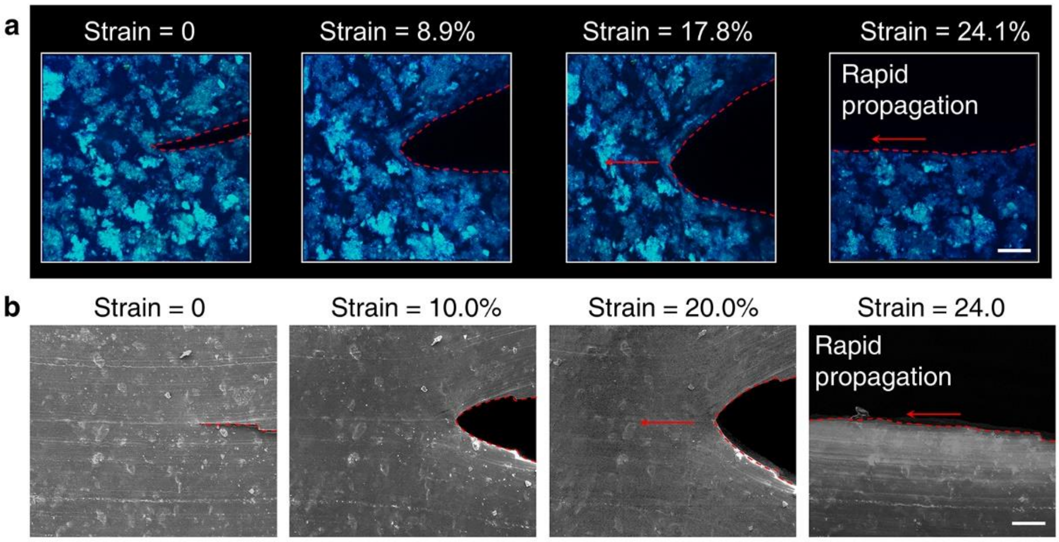

Supplementary Figure 24. In situ CFM characterization (**a**) and SEM characterization (**b**) of crack propagation of the PDMS-MMT-R nanocomposite. Both the CFM and SEM characterization show a rigid unstable crack propagation. The CFM images reveal the distribution of MMT-PVA particles clearly. Scale bar: 100 μm.
